# Loss of FGFR4 promotes the malignant phenotype of PDAC

**DOI:** 10.1038/s41388-022-02432-5

**Published:** 2022-08-13

**Authors:** Sabrina D’Agosto, Francesco Pezzini, Lisa Veghini, Pietro Delfino, Claudia Fiorini, Gael D. Temgue Tane, Anais Del Curatolo, Caterina Vicentini, Giorgia Ferrari, Davide Pasini, Silvia Andreani, Francesca Lupo, Elena Fiorini, Giulia Lorenzon, Rita T. Lawlor, Borislav Rusev, Antonia Malinova, Claudio Luchini, Michele Milella, Elisabetta Sereni, Antonio Pea, Claudio Bassi, Peter Bailey, Aldo Scarpa, Emilio Bria, Vincenzo Corbo

**Affiliations:** 1 Department of Diagnostics and Public Health, University and Hospital Trust of Verona, Verona, Italy; 2 ARC-Net Research Centre, University and Hospital Trust of Verona, Verona, Italy; 3 Department of Medicine, Section of Oncology, University and Hospital Trust of Verona, Verona, Italy; 4 Department of Surgery, University and Hospital Trust of Verona, “Pancreas Institute”, Verona, Italy; 5 Institute of Cancer Sciences, University of Glasgow, Glasgow, UK; 6 Cancer Research UK Beatson Institute, Glasgow, UK; 7 Department of General Surgery, University of Heidelberg, Heidelberg, Germany; 8 Comprehensive Cancer Center, Fondazione Policlinico Universitario Agostino Gemelli IRCCS, Rome, Italy; 9 Section of Medical Oncology, Department of Translational Medicine and Surgery, Università Cattolica del Sacro Cuore, Rome, Italy; 10 Present Address: Human Technopole, Milan, Italy

**Keywords:** Pancreatic cancer, TOR signalling

## Abstract

Transcriptomic analyses of pancreatic ductal adenocarcinoma (PDAC) have identified two major epithelial subtypes with distinct biology and clinical behaviours. Here, we aimed to clarify the role of FGFR1 and FGFR4 in the definition of aggressive PDAC phenotypes. We found that the expression of *FGFR4* is exclusively detected in epithelial cells, significantly elevated in the classical PDAC subtype, and associates with better outcomes. In highly aggressive basal-like/squamous PDAC, reduced *FGFR4* expression aligns with hypermethylation of the gene and lower levels of histone marks associated with active transcription in its regulatory regions. Conversely, *FGFR1* has more promiscuous expression in both normal and malignant pancreatic tissues and is strongly associated with the EMT phenotype but not with the basal-like cell lineage. Regardless of the genetic background, the increased proliferation of FGFR4-depleted PDAC cells correlates with hyperactivation of the mTORC1 pathway both in vitro and in vivo. Downregulation of *FGFR4* in classical cell lines invariably leads to the enrichment of basal-like/squamous gene programs and is associated with either partial or full switch of phenotype. In sum, we show that endogenous levels of *FGFR4* limit the malignant phenotype of PDAC cells. Finally, we propose FGFR4 as a valuable marker for the stratification of PDAC patients.

## Introduction

Pancreatic ductal adenocarcinoma (PDAC) is a malignancy arising from the exocrine pancreatic epithelium and is the deadliest cancer worldwide [1, 2]. The abysmal prognosis of PDAC is contributed by late diagnosis, its complex biology, and lack of effective treatments. The molecular taxonomy of PDAC has been redefined by multiple studies that have used transcriptomic profiling to analyse bulk tissues, cell lines, and microdissected epithelia [3–7]. All classifications have highlighted the existence of two major subtypes based on characteristics of the neoplastic epithelium, namely the classical/progenitor and the basal-like/squamous subtypes. The classical/progenitor PDAC subtype is often regarded as the default pancreatic cancer subtype [5, 8] as it is characterised by the expression of transcription factors involved in specification and maintenance of pancreatic cell fate [3, 9, 10]. Conversely, the basal-like/squamous subtype is associated with loss of pancreatic endodermal identity, and expression of the master regulator of basal-like cells program ΔNp63 [3, 7, 11]. This subtype shows a significantly worse survival outcome and is enriched for inactivation of *TP53* and chromatin regulators, including *ARID1A* and *KDM6A* [3, 12]. Preclinical data have showed that the two subtypes differ also in the response to chemotherapy [6, 10, 13], to agents targeting the cell cycle [14], and further display unique metabolic vulnerabilities [15]. While there is some clinical evidence for the predictive value of transcriptomic classifications [10, 16, 17], clinical investigations are ongoing to conclusively demonstrate the relevance of subtype-specific treatments. Previous works have elegantly demonstrated the causative involvement of pancreatic and endodermal transcription factors (e.g. GATA6, HNF1A, HNF4A) into the maintenance of subtype-specific gene programs [10, 15, 18, 19]. In particular, the emergence of the basal-like/squamous program is almost invariably associated with the loss of expression of transcription factors regulating pancreatic cell fates. Cell fate maintenance is also ensured by the signalling of growth factors through receptor tyrosine kinases (RTKs) [20, 21]. Following activation, RTKs transmit intracellular signals of varying qualities and quantities that alter the transcriptional landscape of a cell [20]. The fibroblast growth factors (FGFs) to Fibroblast growth factor receptors (FGFRs) axis is reportedly involved in the maturation of pancreatic cells from the endoderm [22–25]. Here, we explored the involvement of FGFRs in the definition of molecular subtypes of PDAC. We report the downregulation of *FGFR4* in PDAC showing basal-like/squamous features. Integrating the analysis of transcriptomic, methylation, and chromatin accessibility datasets from patient-derived tissue specimens and cultures, we shed light on the mechanisms leading to downregulation of *FGFR4* in basal-like/squamous tumours. Conversely, we found that elevated expression of *FGFR1* is a functional marker of Epithelial to Mesenchymal Transition (EMT) rather than of the basal-like/squamous subtype. To elucidate whether *FGFR4* is cause or consequence of more aggressive PDAC subtypes, we targeted the receptor using RNA interference approaches in both monolayer cell cultures and patient-derived organoids to show that its loss accelerates cell proliferation and in vivo growth regardless of the genetic and transcriptomic background of the models. Mechanistically, loss of *FGFR4* was associated with increased fluxes through the mTORC1 pathway and accordingly increased protein synthesis. Downregulation of *FGFR4* in classical/progenitor cell lines invariably led to the enrichment of basal-like/squamous gene programs which was associated with either partial of full switch of phenotype. Overall, our data provide direct evidence that the loss of FGFR4 promotes aggressive phenotypes of PDAC.

## Results

### FGFR4 is associated with the classical phenotype of PDAC

**Fig. 1:**
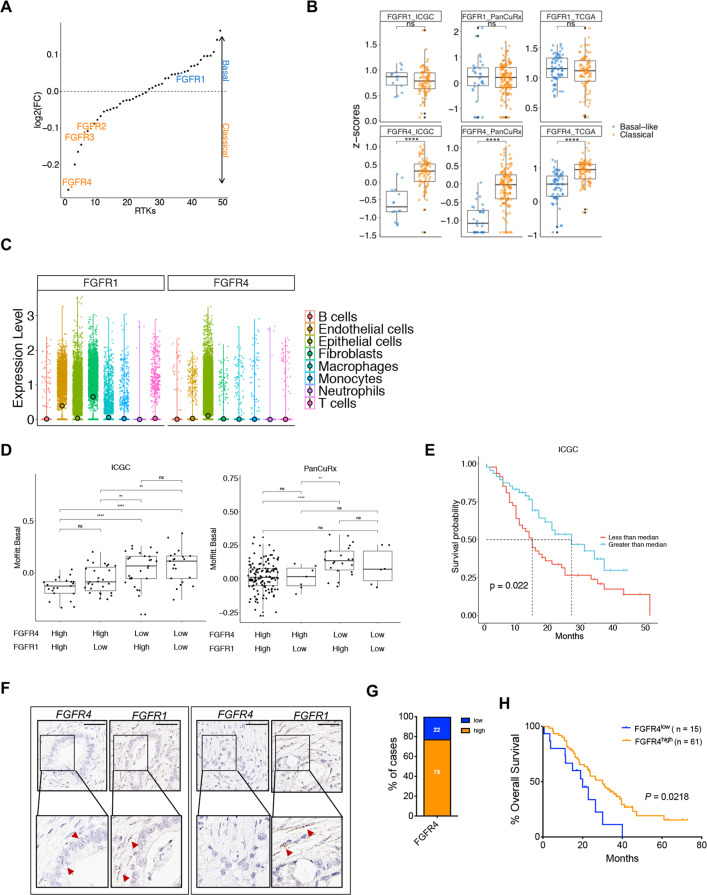
FGFR4 is differentially expressed in PDAC molecular subtypes. **A** Receptor Tyrosine Kinases (RTKs) expression in basal-like and classical subtypes. The scatter plot shows the expressed RTK ranked by their mean log2 fold change in basal-like versus classical for samples of the TCGA cohort [4]. Highlighted: *FGFR1, FGFR2, FGFR3, FGFR4*. **B** Boxplot of *FGFR1* (left) and *FGFR4* (middle) *Z*-scores stratified by the Moffitt subtypes[7] in the TCGA [4], ICGC [3], and PanCuRx [5] datasets. ****p < 0.0001; and ns, not significant as determined by Wilcoxon test. **C** Violin plots of the normalised expression of *FGFR1* (left panel) and *FGFR4* (right panel) in each annotated cell cluster from the integration of four different scRNA-seq datasets of PDAC tissues [5, 26–28] (see methods). **D** GSVA score (using ssgsea method) for the basal-like signature [7] for each sample of the ICGC [3] and PanCuRx [5] cohort according to the expression of *FGFR1* and *FGFR4*. **p < 0.01; ****p < 0.0001; and ns, not significant as determined by Wilcoxon test. **E** Kaplan–Meier plot comparing the overall survival of patients from the ICGC cohort [3] (*n* = 96) according to the expression of *FGFR4*. p, Log-rank (Mantel–Cox) test. **F** Representative ISH images showing expression of *FGFR4* and *FGFR1* in two different pancreatic cancer tissues. Scale Bar, 50 µm. Insets show magnification of selected areas, and red arrowheads indicate either epithelial or stromal cells. **G** Stacked bar plot showing the percentage of PDAC tissues (*n* = 97) with either low (blue) or high (orange) expression of *FGFR4*. **H** Kaplan–Meier plot comparing the overall survival of patients according to FGFR4 ISH status. p, Log-rank (Mantel–Cox) test.

Receptor-Tyrosine Kinases (RTKs) initiated signaling is critical to cell fate determination [20, 21]. To investigate the functional relevance of RTKs in determining PDAC cell lineages and promoting its malignant phenotype, we started by exploring transcriptomic data of human PDAC specimens from the TCGA consortium [4] and found the selective enrichment of *FGFR4* in the classical subtype (Fig. 1A, B). We reproduced this finding in transcriptomic data from two additional cohorts [3, 5] (Fig. 1B). While relatively enriched in basal-like PDAC (Fig. 1A), the expression of *FGFR1* did not significantly discriminate basal-like from classical tumours in the 3 PDAC cohorts [3–5] investigated (Fig. 1B). Next, we explored available single-cell RNA-Seq (scRNA-Seq) data of human PDAC tissues to localise the expression of both *FGFR1* and *FGFR4*. We integrated four PDAC scRNA-seq data sets [5, 26–28] using Harmony [29] and performed cell type annotation to find that *FGFR4* is predominantly expressed by epithelial cells, while *FGFR1* could be detected in epithelial, stromal (fibroblasts, endothelial cells), and immune cells (i.e. macrophages) (Fig. 1C). Next, we explored scRNA-seq data from normal pancreas [30–32] (Supplementary Fig. 1A) and found that the expression of *FGFR4* is mostly restricted to the epithelial cells while *FGFR1* is detectable in many cell types. In situ hybridisation (ISH) analyses of normal pancreatic tissues confirmed scRNA-seq data (Supplementary Fig. 1B). To better understand the association of epithelial FGFRs expression with PDAC molecular subtypes, we then focused on transcriptomic datasets derived from either tissue with high neoplastic cellularity (i.e. ICGC [3], average qPure score > 60%) or microdissected neoplastic epithelia (i.e. PanCuRx [5]). First, we divided samples of the two cohorts in four different groups based on *FGFR1* and *FGFR4* expression statuses (see methods and Supplementary Fig. 1C). To explore the association of *FGFR1* and *FGFR4* with the aggressive basal-like and squamous molecular subtypes as defined by Moffitt [7] and Bailey [3] (Fig. 1D, Supplementary Fig. 1D), we calculated the basal-like and squamous signature scores for each sample of the ICGC and the PanCuRx cohorts [3, 5] (see methods). Tumours with low levels of *FGFR4* showed the highest basal-like/squamous scores regardless of the *FGFR1* status (either high or low); indeed, the signature scores between the *FGFR4*^*low*^*FGFR1*^*high*^ and *FGFR4*^*low*^*FGFR1*^*low*^ were not significantly different (Fig. 1D and Supplementary Fig. 1D). Accordingly, all the *FGFR4*^high^ tumours of the ICGC cohort classified as classical, while only 9.7% (13/134) of the *FGFR4*^high^ tumours of the PanCuRx were defined as basal-like (Supplementary Fig. 1E). These results were substantially similar to those obtained when tumours of the two cohorts were separated based on the expression level of the known classical driver *GATA6* (Supplementary Fig. 1F). When using the molecular classification proposed in the PanCuRx study [5], the group of *FGFR4*^*low*^ tumours was highly enriched for basal subtypes (Supplementary Fig. 1G). Reduced *FGFR4* expression in PDAC tissues from the ICGC [3] was associated with inferior overall survival (Fig. 1E). Conversely, the levels of *FGFR1* had no prognostic significance in different PDAC cohorts (*n* = 3, data not shown). To corroborate these findings, we used ISH to evaluate the expression of *FGFR1* and *FGFR4* in a cohort of 106 human pancreatic tissues from treatment naïve patients (Fig. 1F, Supplementary Fig. 2A, and Supplementary Table 1). A total of 97 tissues were suitable for evaluation of both FGFRs. We confirmed that *FGFR4* is expressed by epithelial cells while *FGFR1* is prominent in stromal elements (Fig. 1F and Supplementary Fig. 2A). Low expression of *FGFR4* was observed in 23% (22/97) of cases (Fig. 1G) and was significantly enriched in high grade tumours (Supplementary Fig. 2B). Furthermore, *FGFR4* was never detected in poorly differentiated areas while detectable with variable degree of expression in well-differentiated tumour glands (Supplementary Fig. 2C). Finally, low expression of *FGFR4* identified patients showing inferior overall survival in our cohort (Fig. 1H). To identify suitable human models for genetic manipulation of the two FGFRs, we screened an initial array of cell lines (*n* = 6) and Patient-derived Organoids (*n* = 5). In accordance with our results, ISH analyses of 6 human PDAC cell lines revealed high *FGFR4* in cells displaying an epithelial phenotype (Supplementary Fig. 2D–F), and higher levels of FGFR1 in mesenchymal-like cells showing expression of ZEB1 and/or Vimentin (Supplementary Fig. 2D–F). For PDOs, RNA-seq was used to classify cultures as either classical or basal-like using single-sample Gene Set Variation Analysis (ssgsea method) [33]. Of note, the organoid culture with the highest basal-like/squamous identity (PDA9-O) showed the lowest expression of FGFR4 (Supplementary Fig. 2G), thus representing a model for FGFR4^low^ basal-like tumours.

### FGFR1 is a functional marker of EMT in PDAC

**Fig. 2:**
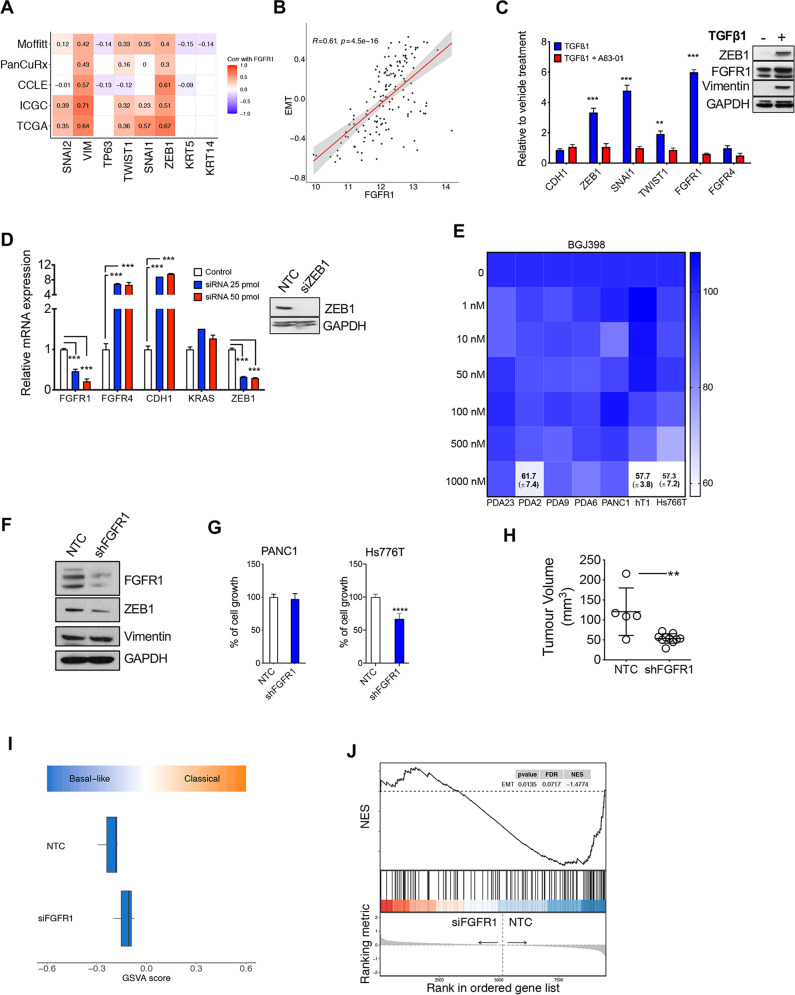
FGFR1 is a functional marker of EMT in PDAC. **A** Heatmap showing correlation (Spearman’s correlation) between *FGFR1* and the squamous lineage markers, and the EMT genes in five different transcriptomic datasets [3–5, 7, 34]. All annotated boxes, *p* < 0.001 **B** Scatter plot showing the positive correlation between *FGFR1* mRNA expression and the Epithelial-to-Mesenchymal Transition (EMT) signature score from MsigDB for the TCGA cohort [4]. **C** Changes in the expression level of the indicated genes in hM1 cell line treated with 5 ng/mL of TGFβ1 alone or in combination with 500 nM of TGFβ1 inhibitor (A83-01) for 48 h. On the right, immunoblot analysis of ZEB1, FGFR1, and Vimentin in whole-cell lysates from hM1 treated with 500 nM TGFβ1 for 48 h. GAPDH was used as loading control. **D** Changes at 48 h in the expression level of the indicated genes in the Hs766T cell line transfected with either mock control or two different concentrations of siRNAs targeting ZEB1. On the right, immunoblot analysis of ZEB1 in whole-cell lysates of PANC1 transfected with non-targeting control (NTC) or 25 pmol of siRNA against *ZEB1*. GAPDH was used as loading control. In (**C** and **D**), results shown as mean ± SD of three replicates. **p < 0.01; ***p < 0.001 as determined by Student*’*s *t* test. **E** Cell viability of PDAC monolayer cultures treated with BGJ398 (*n* = 7 cell cultures) as indicated. Data are displayed as heatmap of the percentage of inhibition at each dose of the drugs and presented as mean of three independent experiments. **F** Immunoblot analysis of FGFR1, ZEB1, and Vimentin in whole-cell lysates of PANC1 cells stably expressing the control vector (NTC) or the shRNA targeting FGFR1 (shFGFR1). GAPDH was used as loading control. **G** Relative growth (as percentage of cell proliferation) of PANC1 and Hs766T cells stably transduced with either the control vector (NTC) or the vector targeting FGFR1. Data presented are means ± SD of three biological replicates. **H** Scatter dot plot showing differences in tumour volumes between tumour-bearing mice transplanted with PANC1/NTC (*n* = 5 mice) or PANC1/shFGFR1 (*n* = 10 mice). Tumour volumes were measured by ultrasound 5 weeks after transplantation. **p < 0.01 as determined by Student’s *t* test. **I** Boxplots of GSVA score (based on ssgsea method) for the Classical and the Basal-like signatures calculated for the Hs766T cell line transfected with non-targeting control or siRNA against *FGFR1*. **J** GSEA plot evaluating the EMT signature upon *FGFR1* knockdown in Hs766T cell line.

The interrogation of the genomic data from the ICGC [3] and TCGA [4] cohorts in cBioportal (https://www.cbioportal.org) revealed no recurrent genetic alteration affecting *FGFR1* in PDAC (Supplementary Fig. 3A, B). We then looked at the correlation between *FGFR1* and the established markers/drivers of PDAC cell phenotypes. In all the interrogated transcriptomic datasets (*n* = 5) [3–5, 7, 34], *FGFR1* expression positively correlated with the expression of EMT genes, in particular with the master regulator *ZEB1*, yet not with expression of squamous lineage genes (*TP63*, *KRT5*, *KRT14*) (Fig. 2A). To corroborate the suggested link with EMT, we found a significant positive correlation between the Hallmark EMT signature and *FGFR1* in samples from the TCGA cohort [4] (Fig. 2B). Next, we investigated whether induction of EMT or downregulation of its master regulator ZEB1 would affect expression of FGFR1. Following 48 h of TGFβ1 treatment, the SMAD4-proficient cell line hM1 displayed significant elevation in the expression of several EMT markers/drivers as well as of FGFR1 both at mRNA and protein levels (Fig. 2C). The mRNA levels of *CDH1* and *FGFR4* were only moderately affected by the treatment. Furthermore, the transient downregulation of ZEB1 in the PDAC cell line showing the more prominent mesenchymal-like phenotype (i.e. PANC1) led to a significant reduction in the expression of FGFR1, while increasing expression of *FGFR4* and the classical gene *CDH1* (Fig. 2D). Then, we sought to assess the functional consequences of FGFR1 inhibition in PDAC. First, we treated 7 human PDAC cell lines with increasing concentrations of the FGFR1 inhibitor BGJ398 [35] and measured cell viability using an ATP-based assay (Fig. 2E). Four out of the seven cell lines tested were primary cell lines (PDA2, PDA9, PDA23, PDA6), which were classified as either basal-like or classical based on RNA-Seq data (Supplementary Fig. 3D and data not shown). BGJ398 demonstrated poor activity and modestly affected proliferation of PDA2, hT1, and of Hs766T only at the highest dose (1 µM) (Fig. 2E). Then, we performed genetic depletion of FGFR1 by RNA interference approaches. Stable silencing of FGFR1 in PANC1 was associated with reduced protein expression of the EMT driver ZEB1 but not of the marker Vimentin (Fig. 2F). In vitro proliferation of Hs766T, but not of PANC1, was significantly reduced following downregulation of *FGFR1* (Fig. 2G). However, orthotopically transplanted PANC1 cells deficient for FGFR1 generated smaller tumours compared to parental cells transduced with the empty vector (Fig. 2H), thus suggesting cell extrinsic effects of FGFR1 downregulation in this cell line. In addition, we performed RNAi-based competition assay in the basal-like PDA9-O which displayed the highest protein levels of FGFR1 among the organoid cultures investigated. We observed the progressive drop-out of the vector targeting *FGFR1* (Supplementary Fig. 3E), suggesting that FGFR1 is advantageous for the proliferation of this organoid culture. We next sought to explore whether loss of *FGFR1* in a basal-like background could lead to changes in PDAC cell identity. Using RNAi, we targeted *FGFR1* in the basal-like Hs766T (PDX1^−^CK5^+^, Supplementary Fig. 2E) and performed RNA-Seq analysis (Supplementary Fig. 3F and Supplementary Table 2). GSEA demonstrated that the loss of *FGFR1* led to alteration of genes programs related to cell proliferation and Interferons’ response (Supplementary Fig. 3G, Supplementary Table 3). However, the loss of *FGFR1* did not lead to significant changes in subtype expression signatures and accordingly *FGFR1*-deficient cells were classified as basal-like (Fig. 2I). Conversely, downregulation of *FGFR1* led to the significant reduction of the EMT transcriptional phenotype (Fig. 2J). In sum, our analysis reveals that FGFR1 is a functional driver of EMT in pancreatic cancer.

### FGFR4 is epigenetically downregulated in basal-like/squamous PDAC

**Fig. 3:**
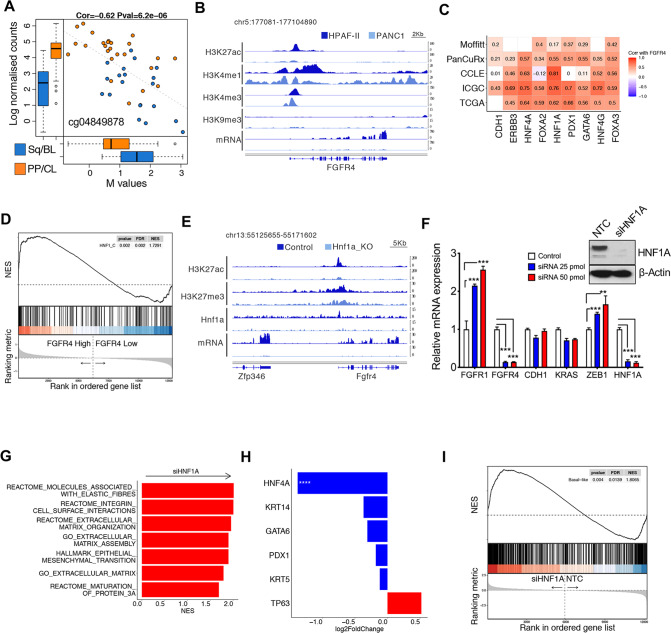
Expression of FGFRs4 and determinants of molecular subtypes. **A** Hypermethylation of *FGFR4* in squamous/basal-like tumours (blue) from the ICGC cohort [3] is concordant with the downregulation of the gene. Indicated is the CpG probe showing the highest correlation. **B** Representative snapshot of the genomic region of *FGFR4* in PANC1 (high-grade, basal-like) and HPAF-II (low-grade, classical) from Diaferia et al. [36] showing histone modifications (H3K27ac, H3K4me1, H3K4me3, H3K9me3) and RNA-seq data. **C** Heatmap showing correlation (Spearman’s correlation) between *FGFR4* and the expressed endodermal transcription factors and epithelial genes in five different transcriptomic datasets [3–5, 7, 34]. All annotated boxes, *p* < 0.001. **D** GSEA plot evaluating the enrichment of the geneset containing putative HNF1A target genes when comparing *FGFR4* high versus *FGFR4* low tumours of the ICGC cohort [3]. **E** Representative snapshot of the genomic region of *Fgfr4* in mouse pancreatic cells proficient (control) or deficient (Hnf1a_KO) for HNF1A from Klasniz et al. [19] showing histone modifications (H3K27ac, H3K27me3), HNF1A occupancy, and RNA-seq data. **F** Changes in the expression levels of the indicated genes in the HPAF-II cell line transfected with either mock control or two different concentrations of siRNA against *HNF1A*. Results are shown as mean ± SD of three replicates. ***p < 0.001 as determined by Student*’*s *t* test. On the right, immunoblot analysis of HNF1A in whole-cell lysates of HPAF-II transfected with either non-targeting control (NTC) or 25 pmol of siRNA targeting HNF1A. β-Actin was used as loading control. **G** Enrichment of selected pathways upon *HNF1A* knockdown. The GSEA analysis was performed using gene sets from REACTOME, GO, Hallmark, and REACTOME databases in MsigDB library. Displayed gene sets that passed false-discovery rate < 0.05. See also Supplementary Table 5. **H** Expression of classical and basal-like genes in HNF1A-deficient HPAF-II cells (compared to parental cells) from RNA-Seq data. Data are presented as mean ± SD. ****, *p* < 0.0001 by Student *t* test. **I** GSEA plot evaluating the basal-like signature upon depletion of HNF1A in HPAF-II cell lines.

The reduced expression of FGFR4 in aggressive subtypes of PDAC might be due to either genetic or epigenetic mechanisms. First, we explored the genomic data from the ICGC [3] and TCGA [4] cohorts and found no recurrent genetic alterations affecting *FGFR4* (Supplementary Fig. 3A, C). Therefore, we interrogated the available methylation data from the ICGC cohort [3] and found that the downregulation of *FGFR4* in the squamous subtype was associated with hypermethylation of the gene (Fig. 3A). Next, we reanalysed data from Diaferia et al. [36] that used ChIP-Seq to profile marks indicative of either active (e.g. H3K27ac) or inactive (e.g. H3K9me3) chromatin in PDAC cell lines representative of classical and basal-like tumours. We found that the genomic region proximal to the *FGFR4* transcription start site showed differential levels of H3K27ac between basal-like and classical cell lines, with changes that were concordant with higher level of transcripts in the classical cell line (Fig. 3B and Supplementary Fig. 4A). A broader peak of the active enhancer chromatin mark H3K4me1 was observed for the classical cell line in the intron 1 of *FGFR4* gene (Fig. 3B), which has been reported to contain an enhancer region [37]. In keeping with that, *FGFR4* expression positively correlated with the expression of endodermal transcription factors and epithelial genes (*CDH1* and *ERBB3*) (Fig. 3C) in 5 different transcriptomic datasets of PDAC [3–5, 7, 34]. In particular, *FGFR4* showed significant positive correlation with the transcription factor *HNF1A* in all datasets [3–5, 7, 34] (Fig. 3C). Accordingly, putative target genes of HNF1A were significantly enriched in *FGFR4* high tumours of the ICGC cohort (Fig. 3D). To prove direct regulation by the transcription factor, we reanalysed available data from pancreas-specific knockout of *Hnf1a* [19]. In *Hnf1a*-deficient versus proficient pancreatic cells, expression of *Fgfr4* was significantly reduced and this was concordant with genomic regions proximal to the transcription start site of *Fgfr4* showing reduced occupancy by HNF1A and reduced levels of H3K27ac (Fig. 3E). Overall, our analysis strongly suggests that *FGFR4* is a marker of the classical subtype whose expression is epigenetically reduced in basal-like PDAC cells.

### HNF1A blocks EMT in a classical PDAC cell line

To confirm the link between *HNF1A* and *FGFR4* in the human PDAC setting, we manipulated the expression of *HNF1A* by RNAi in HPAF-II, which has been reportedly used as a model of classical PDAC [36, 38]. The successful downregulation of *HNF1A* was associated with the reduced expression of *FGFR4* and increased expression of both *ZEB1* and *FGFR1* (Fig. 3F). Downregulation of *HNF1A* was associated with substantial changes in the transcriptome of the HPAF-II cell line (Supplementary Fig. 4B and Supplementary Table 4) and gene-set enrichment analysis (GSEA) [39] showed the enrichment of several pathways and terms related to extracellular matrix organization/deposition and the EMT (Fig. 3G, and Supplementary Table 5). Of the marker/drivers of PDAC epithelial subtypes, only *HNF4A* was significantly downregulated upon *HNF1A* knockdown (Fig. 3H). In keeping with a recent work [18], *HFN1A* downregulation was not sufficient to drive a full phenotype switch in HPAF-II cell line. Yet, *HNF1A* silencing significantly increased the expression of genes defining the basal-like signature [7] as assessed by GSEA (Fig. 3I).

### Loss of FGFR4 enhances the malignant behaviour of PDAC cells

**Fig. 4:**
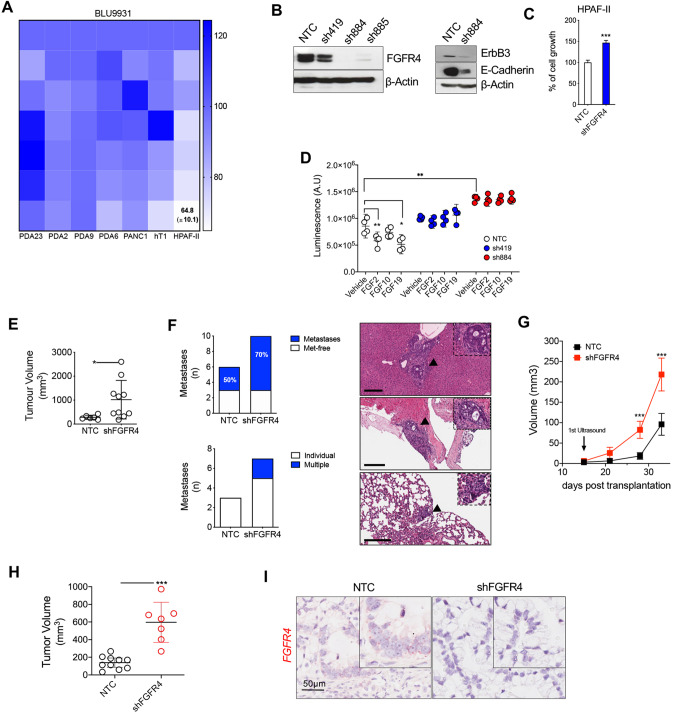
Loss of FGFR4 is associated with an aggressive PDAC biological behaviour. **A** Cell viability of PDAC monolayer cultures treated with BLU3391 (*n* = 7 cell cultures) as indicated. Data are displayed as heatmap of the percentage of inhibition at each dose of the drugs and presented as mean of three independent experiments. **B** Immunoblot analysis of FGFR4, ErbB3, and E-Cadherin (loading control, β-Actin) in HPAF-II stably expressing the control vector (NTC) or the construct targeting FGFR4 (shFGFR4). **C** Relative growth (as percentage of cell proliferation) of HPAF-II cells stably transduced with either the control vector (NTC) or the vector targeting FGFR4 (#884). Data presented are means ± SD of three biological replicates. **D** Proliferation (as total luminescence) of HPAF-II cell line stably transduced with NTC or two different short hairpin RNA against FGFR4 (#419 and #884) following 48 h stimulation with FGF2 (25 ng/mL), FGF10 (100 ng/mL), and FGF19 (100 ng/mL). Data presented as means ± SD (*n* = 3 biological replicates). *p < 0.05; and **p < 0.01 as determined by Student’s *t* test. **E** Scatter dot plot showing differences in tumour volumes between tumour-bearing mice transplanted with HPAF-II/NTC (*n* = 6 mice) and HPAF-II/shFGFR4 (*n* = 10 mice). Tumour volumes were measured by ultrasound 4 weeks after transplantation. *p < 0.05 as determined by Student’s *t* test. **F** Stacked bar plot showing the distribution of mice with or without metastatic lesions in the two different cohort of mice from (**E**) (upper panel). Lower panel, stacked bar plot showing the number of mice with individual or multiple metastatic lesions in the two different cohort from (**E**). On the right, representative Hematoxylin and Eosin staining of metastatic lesions at the liver (top and middle) and at the lungs (bottom) from a mouse bearing multiple metastases. The black arrows indicate the areas shown in the insets. Scale bar, 200 µm. **G** Line graph showing tumour volumes (mm3) of pancreatic masses detected upon the orthotopic injection of 1 × 10^6^ cells from hT3 PDO into immunodeficient mice (*n* = 7). Means ± SD are shown. Mice were screened at 15, 21, 27, 32 days following transplantation. ****p < 0.001 by two-way ANOVA with Sidak’s test for multiple comparison. **H** Scatter plot showing the difference in tumour volumes between mice transplanted with PDA9 organoids either transduced with control vector (*n* = 10 mice) or with shFGFR4 (*n* = 7 mice). Tumour volumes were measured by ultrasound 4 weeks after transplantation. ***p < 0.01 as determined by Student’s *t* test. **I** Representative in situ hybridisation staining of *FGFR4* in PDA9-O in mice from (**H**) and showing loss of *FGFR4* in vivo. Scale bar, 50 μm.

To evaluate the functional relevance of FGFR4 in dictating PDAC phenotypes, we performed genetic and pharmacological perturbation experiments (Fig. 4 and Supplementary Fig. 4). Cell viability of established cell lines and primary PDAC cells (*n* = 7) was not significantly inhibited by the continuous treatment with the FGFR4 inhibitor BLU9331 [40] (FGFR4i) (Fig. 4A). Of note, low doses of FGFR4i slightly increased the proliferation of some PDAC cell lines. Also considering the dose-response analysis of PDAC cells with the FGFR1 inhibitor BGJ398 (Fig. 2E), our results show that pharmacological inhibition of FGFRs is not a viable strategy to reduce PDAC cells proliferation. Next, we targeted *FGFR4* in the classical cell line HPAF-II using three different shRNAs (Fig. 4B). *FGFR4* was successfully downregulated by all shRNAs with the #884 and the #419 showing the highest and lowest efficiency, respectively (Fig. 4B). The majority of results were obtained using the two most efficient shRNA, namely #884 and #885. Loss of *FGFR4* in HPAF-II cell line was associated with downregulation of the epithelial markers E-Cadherin and ERBB3 (Fig. 4B) and increased in vitro proliferation (Fig. 4C). Interestingly, downregulation of FGFR4 expression altered the proliferative response to three different FGF ligands (Fig. 4D). In particular, continuous treatment with FGF2 and FGF19 significantly reduced the proliferation of control HPAF-II cell line but did not affect the proliferation of cells displaying downregulation of *FGFR4* (Fig. 4D). Furthermore, we orthotopically transplanted HPAF-II transduced with non-targeting and targeting vectors into immunocompromised mice and monitored tumour growth for 4 weeks. At the experimental endpoint, loss of *FGFR4* in HPAF-II cell line generated larger tumours with increased number of mitotic figures (Fig. 4E, and data not shown), and significantly increased the metastatic burden at the lungs and the liver (Fig. 4F). In agreement with that, the group of *FGFR4*^*low*^ tumours of the PanCuRx cohort [5] was significantly enriched for metastases (Supplementary Fig. 4C). The in vivo pro-tumorigenic effect driven by the loss of *FGFR4* was further validated in organoid-based xenografts with the classical PDO hT3 (Supplementary Fig. 2G). Immunodeficient mice we orthotopically transplanted with equal number of cells from either parental PDOs (*n* = 7) or cultures stably transduced with the shRNA targeting *FGFR4* (*n* = 7) (Supplementary Fig. 4D). We monitored tumour growth with high-contrast ultrasound imaging starting at day 15 post transplantation. In agreement with the results obtained with established cell lines, PDOs expressing high levels of FGFR4 displayed reduced proliferation in vivo (Fig. 4G) and less efficient engraftment rate at earlier time points as only 1 out of 7 transplanted mice showed a detectable mass at day 15 as opposed to the FGFR4 deficient PDO (5/7 mice with detectable masses). Finally, we downregulated *FGFR4* in the organoid model of FGFR4^low^ basal-like tumours (PDA9-O) prior to in vivo transplantation in immunocompromised hosts. At the endpoint, the tumour masses were significantly larger in animals transplanted with FGFR4 deficient cells (Fig. 4H). ISH analyses of transplanted tissues confirmed the downregulation of *FGFR4* as opposed to control mice (Fig. 4I and Supplementary Fig. 4E). To further generalise our results, we transiently downregulated *FGFR4* in four PDAC cell lines (including HPAF-II) and observed increased proliferation regardless of the molecular subtype of the cells (Supplementary Fig. 4F). In agreement with that, GSEA comparing the transcriptomes of *FGFR4*^*low*^ versus *FGFR4*^*high*^ tumours in the ICGC cohort revealed the significant enrichment of terms related to “cell proliferation” and “cell cycle” in *FGFR4*^*low*^ PDAC (Supplementary Fig. 4G). Overall, these data suggest that endogenous levels of FGFR4 limit the malignant phenotype of PDAC regardless of the genetic background of the cells.

### Loss of FGFR4 is associated with increased basal-like/squamous features in PDAC

**Fig. 5:**
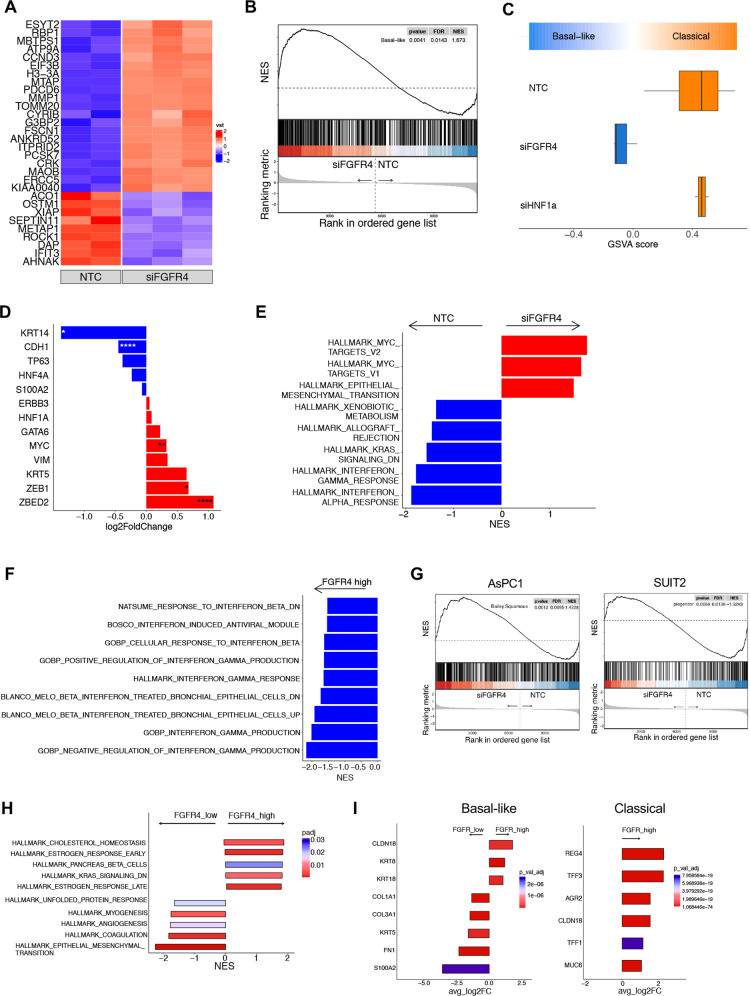
FGFR4 loss leads to the expression of basal-like/squamous programs in PDAC. **A** Heatmap showing changes in the expression pattern of the 30 most differentially expressed genes in the comparison between: control and FGFR4 knock-down (siFGFR4). *Z*-scores derived from DESeq2-VST transformed counts. See also Supplementary Table 6. **B** GSEA plot evaluating the Basal-like signature upon *FGFR4* knockdown in HPAF-II cell line. **C** Boxplots of GSVA score (based on the ssGSEA method) evaluating the Basal-like and Classical signatures [7] upon FGFR4 knockdown. **D** Expression of classical and basal-like genes in FGFR4-deficient HPAF-II cells (compared to parental cells) from RNA-Seq data. Data are presented as mean ± SD.*p < 0.05; **p < 0.01; and ****p < 0.0001 by Student *t* test. **E** Enrichment of selected pathways (GSEA) when comparing HPAF-II proficient (NTC) and deficient (siFGFR4) cells. GSEA was performed using gene sets from Hallmark database in MsigDB library. Displayed gene sets that passed false-discovery rate < 0.05. **F** Enrichment of Interferon related pathways when comparing *FGFR4* low versus *FGFR4* high tumours of the PanCuRx cohort [5]. GSEA was performed using gene sets from databases in MsigDB library. Displayed gene sets that passed false-discovery rate < 0.05. **G** GSEA plot evaluating the Bailey_Squamous (left panel) and the Bailey_Progenitor (right panel) signatures upon *FGFR4* knockdown in AsPC1 and in SUIT2 cell lines, respectively. **H** Enrichment of selected pathways (GSEA) when comparing *FGFR4* high versus *FGFR4* cells from the scRNA-Seq dataset of Peng et al. [27]. See also Supplementary Fig. 5I. GSEA was performed using gene sets from Hallmark database in MsigDB library. **I** Expression of classical and basal-like/squamous genes in *FGFR4* high cells (compared to *FGFR4* low cells) displaying either a basal-like (left panel) or a classical (right panel) phenotype.

We next sought to explore whether loss of *FGFR4* in a classical background could lead to changes in PDAC cell identity. *FGFR4* knockdown in HPAF-II cells led to substantial changes in the transcriptome of this classical cell line (Supplementary Table 6 and Fig. 5A). Similarly, *FGFR4* knockdown led to profound changes in the expression of genes that drive the signatures of molecular subtypes of PDAC (Fig. 5B) and deficient cells were classified as basal-like (Fig. 5C) suggesting that, in this cell line, FGFR4 is a superior barrier to the expression of ectopic gene programs (i.e. basal-like) compared to HNF1A. We then looked at changes in the expression of markers/drivers of subtype following *FGFR4* silencing and found reduced expression of *CDH1* (Fig. 5D), which is consistent with the reduction of E-cadherin (protein level) following stable knockdown (Fig. 4B). Of transcription factors previously associated with the basal-like/squamous lineage, ZBED2 showed the most prominent upregulation upon FGFR4 silencing (Fig. 5D). ZBED2 has been recently demonstrated to antagonise the action of interferon regulatory factor 1 (IRF1), to be selectively enriched in squamous tumours, and to prevent the growth arrest induced by Interferon in PDAC cells [41]. Accordingly, silencing of *FGFR4* led to downregulation of gene sets related to interferon pathway in HPAF-II (Fig. 5E, Supplementary Table 7) while increasing for the expression of gene programs related to c-Myc activity and EMT. To corroborate our findings, we interrogated RNA-seq from patients’ tissues and found that ZBED2 is the gene whose expression is more prominent in *FGFR4* low tumours of the ICGC cohort (Supplementary Fig. 5A). Furthermore, GSEA comparing the transcriptomes of *FGFR4*^*low*^ versus *FGFR4*^*high*^ tumours in both the ICGC and PanCuRx cohorts revealed the significant downregulation of terms related to “Interferon” in *FGFR4*^*low*^ PDAC (Fig. 5F and Supplementary Fig. 5B). To further generalise our results and understand whether the effect of *FGFR4* downregulation on PDAC cell lineages is context dependent, we targeted the receptor by RNAi in two additional models of classical/progenitor (i.e. epithelial) PDAC: AsPC1 and SUIT2. These two cell lines have been extensively used to identify the molecular barriers to the expression of basal-like/squamous programs in PDAC [9, 38] and display high expression of FGFR4 (Supplementary Fig. 5C). Differently from HPAF-II, the forced downregulation of FGFR4 in these two models did not lead to a full switch of phenotype (Supplementary Fig. 5C). Nevertheless, the loss of FGFR4 led to the reduction of classical/progenitor programs and of the epithelial marker E-cadherin, the enrichment of transcriptional signatures of basal-like/squamous PDAC and of genesets related to increased proliferation and c-Myc activity (Fig. 5G, Supplementary Fig. 5D–H). While suggesting that the effect on “full subtype switch” is cell-context dependent, our results strongly suggest that FGFR4 limits the expression of gene programs of aggressive (i.e. basal-like/squamous) PDAC. To further ascertain whether *FGFR4* expression is associated with changes in the expression of gene and gene programs of the two PDAC subtypes in human tissues, we interrogated the scRNA-Seq data from Peng et al. [27]. First, we confirmed the enrichment for FGFR4^high^ cells in the epithelial compartment of patients displaying the classical phenotype (Supplementary Fig. 5I). Then, we separated cases with lowest and highest expression of *FGFR4* (Supplementary Fig. 5J) to find that those with elevated epithelial expression of *FGFR4* accordingly displayed elevated expression of classical markers (Supplementary Fig. 5K). Furthermore, *FGFR4*^low^ cells were characterised by gene expression programs of basal-like/squamous subtypes (Fig. 5H), which included EMT related gene sets. Finally, we sorted out basal-like and classical cells from those cases and compared the transcriptome of cells displaying high and low expression of FGFR4. In line with the suggested role for FGFR4 in sustaining the classical phenotype, classical cells with the highest expression of FGFR4 displayed elevated expression of classical genes (Fig. 5I). Similarly, even within basal-like cells those with the highest expression of *FGFR4* displayed elevated level of classical/epithelial PDAC while the ones with reduced expression of the gene showed increased expression of basal-like/squamous markers (Fig. 5I).

### Loss of FGFR4 leads to increased fluxes through the mTORC1 pathway in PDAC cells

**Fig. 6:**
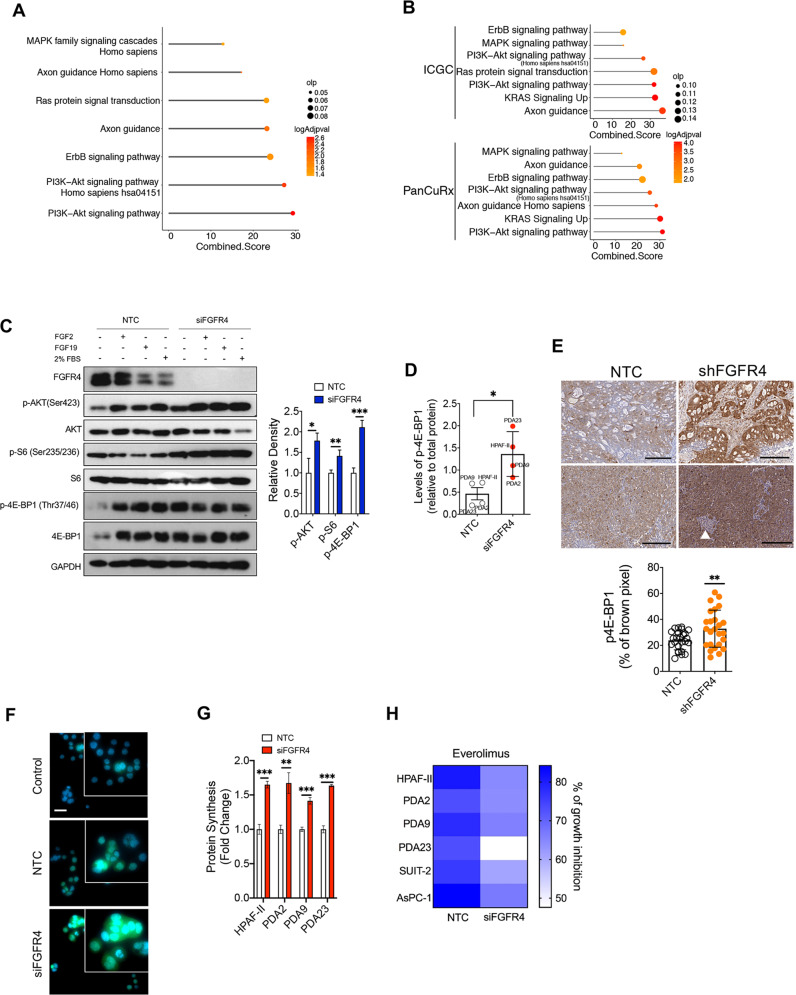
Loss of FGFR4 is associated to hyperactivation of mTORC1 in PDAC. **A** Enrichr pathway analysis of significantly over-represented genes in FGFR4 deficient HPAF-II cells. See also Supplementary Table 8. **B** Enrichr pathway analysis of significantly over-represented genes in *FGFR4*^*low*^ tumours of the ICGC [3] (top) and PanCuRx [5] (bottom) cohorts. See also Supplementary Table 8. **C** Immunoblot analysis in whole-cell lysates of HPAF-II cell line transfected with either control (NTC) or siRNA targeting FGFR4. GAPDH was used as loading control. Quantification of changes in the phosphorylated levels of selected proteins (p-AKT, p-S6, and p-4E-BP1) between NTC and siFGFR4 at baseline is provided in the bar plots on the right (data presented as means ± SD of four biological replicates). *p < 0.05; **p < 0.01; ***p < 0.001 by Student’s *t* test. See also Supplementary Fig. 6D. **D** Scatter plot showing changes in the phosphorylated levels of 4E-BP1 in four different cell cultures (HPAF-II, PDA2, PDA9, PDA23) following transient knockdown of FGFR4. *p < 0.05 by Student’s *t* test. The quantification refers to the immunoblots of (**C**) and Supplementary Fig. 6E–G. **E** Representative images of the immunohistochemical staining for phosho-4E-BP1 in tissues from mice transplanted with either mock (NTC) or shRNA targeting FGFR4. Areas with different histologies were included in the analyses. White arrowhead refers to islet of Langerhans, which stained negative and used as internal control. The quantification is provided in the scatter dot plot (bottom panel) as percentage of brown pixel (mean intensity/nuclei). A minimum of five field of visualisation (FOVs, 20X areas) per mouse (5 mice/cohort) was analysed. Scale bars, 200 µm. **F** Immunodetection of DAPI (blue), and newly synthesised protein (green) in cycloheximide-treated HPAF-II cells (top), and HPAF-II transfected with control (NTC, middle panel) and siRNA targeting FGFR4 (bottom panel). Scale bar, 20 μm. **G** Quantitative Click-iT HPG Alexa Fluor 488 immunofluorescence labeling showed a significant increase in nascent protein synthesis following the silencing of FGFR4 in different monolayer cultures. Data presented as means ± SD (*n* = 3 biological replicates per cell culture). **p < 0.01; and ***p < 0.001 as determined by Student’s *t* test. **H** Cell viability of PDAC monolayer cultures transfected with either mock or siRNA targeting FGFR4 for 48 h and then challenged with 1 µM of the mTORC1 inhibitor Everolimus for 48 h (see methods). Data are displayed as heatmap of the percentage of inhibition relative to the untreated control and presented as mean of three independent experiments.

Next, we used Enrichr [42] on gene expression data to infer signalling pathways’ dysregulation that might explain the increased aggressiveness observed in PDAC cells upon depletion of FGFR4 (Supplementary Table 8). We found the significant enrichment of gene sets related to the PI3K/AKT, ERBB, Axon guidance, and RAS signalling pathways in PDAC cells depleted for *FGFR4* (Fig. 6A, Supplementary Table 8) as well as in the *FGFR4*^*low*^ tumours of both the ICGC [3] and PanCuRx [5] cohorts (Fig. 6B, Supplementary Table 8). First, we evaluated the effect of FGFR4 downregulation on the main signaling activated through the stimulation of FGFRs, the MAPK pathway. Serum-starved HPAF-II and SUIT2 cell lines were stimulated for 20 min with FGF ligands or 2% FBS, which induced the phosphorylation of ERK in both parental and FGFR4-depleted cells (Supplementary Fig. 6A, B). This suggests that the loss of FGFR4 does not compromise signaling through this pathway. Furthermore, the baseline level of phospho-ERK were elevated in both HPAF-II and SUIT2 cell lines upon FGFR4 knockdown, which is in line with the pathway analysis from RNA-seq data. A common single nucleotide polymorphism in the exon 9 of the *FGFR4* gene results in a nucleotide change (G388R), which has been shown to enhance STAT3 activation without altering MAPK/ERK pathway [43]. In our cohort, 7 out of 12 PDAC models carry the variant (see material and methods section). In line with a previous report [43], the polymorphic allele does not explain differences in *FGFR4* transcript levels between cell lines (data not shown). Then, we tested the effect of *FGFR4* knockdown on the activation of STAT3 in cell lines either wild-type (AsPC1) or carrying the polymorphic allele (HPAF-II and SUIT2). In line with the literature [43], the depletion of FGFR4 in SUIT2 and HPAF-II cell lines reduced the baseline and stimulated activation of STAT3 (Supplementary Fig. 6C). Conversely, the downregulation of FGFR4 in AsPC1 cell line did not dramatically affected the activation of STAT3, while consistently driving increased fluxes through MAPK at baseline (Supplementary Fig. 6C). Next, we sought to validate the inferred activation of the PI3K/AKT/mTOR pathway, which resulted the most dysregulated pathway in both cell lines and tissues at transcriptomic level (Fig. 6A, B).

We assessed the baseline and stimulated activation of the PI3K/AKT/mTOR pathway through the evaluation of the phosphorylated levels of AKT and of the downstream mTORC1 effectors (the inhibitory eIF4E-binding protein 4E-BP1, and the substrate of the S6 Kinase). Cells were serum-starved and then stimulated with FGF2, FGF19, and 2% of Fetal-bovine serum (FBS). In parental HPAF-II cell line, 20 min stimulation with either FGF ligands or FBS led to activation of AKT (Ser473), to increased phosphorylation of 4E-BP1, but not to increased phosphorylation of the S6K1 substrate S6 (Fig. 6C and Supplementary Fig. 6D). Strikingly, loss of FGFR4 in serum-starved condition was associated to increased activation of AKT, of S6K and phosphorylation of 4E-BP1 (Fig. 6C). As compared to the parental cell line, HPAF-II lacking FGFR4 showed higher induction of phosphorylated level of AKT, but no further increase in the phosphorylation of 4E-BP1, following stimulation with either FGFs or serum (Fig. 6C and Supplementary Fig. 6D). Overall, these data suggest that loss of *FGFR4* in HPAF-II cell line increases the flux through the mTORC1 pathway in particular through the inhibition of the translational repressor 4E-BP1, which is known to be required for mTORC1-dependent regulation of proliferation in mammalian cells [44]. We replicated these results in five additional cell lines (PDA2-9-23, SUIT2, and AsPC1) by showing that the loss of FGFR4 primarily increases the phosphorylation of 4E-BP1 at baseline (Fig. 6D and Supplementary Fig. 6E–H). In keeping with this, we found increased levels of phosphorylated 4E-BP1 in pancreatic cancer tissues from mice transplanted with HPAF-II stably expressing shFGFR4 (Fig. 6E). Given that mTORC1 is the master regulator of protein synthesis [45], we measured changes in protein synthesis following FGFR4 knockdown in four different PDAC cell lines (see methods). Loss of FGFR4 significantly increased total protein synthesis (Fig. 6F, G) and coherently gene expression terms related to translational initiation were significantly enriched following *FGFR4* knockdown (Supplementary Fig. 6I). Finally, we reasoned that if elevation of mTORC1 pathway activity was primarily responsible for the increased proliferation of PDAC cells upon FGFR4 depletion, then pharmacological inhibition of the pathway could represent a viable strategy to reverse this phenotype. Indeed, we found that, compared to the vehicle-treated cells, the treatment with the mTORC1 inhibitor (Everolimus) resulted in a larger inhibition of cell proliferation in FGFR4 depleted cells as opposed to parental cells (Fig. 6H). Overall, our results indicate that loss of *FGFR4* in PDAC is associated to increased mTORC1-driven cell proliferation regardless of the genetic and transcriptomic background of the cells.

## Discussion

Here, we show that the loss of *FGFR4* expression in PDAC is invariably associated with the acquisition of a more aggressive phenotype by cancer cells driven by hyperactivation of the mTORC1 pathway. Conversely, we found that FGFR1 is a functional marker of the EMT. Receptor-Tyrosine Kinases (RTKs) initiated signalling is critical to cell fate determination [20, 21]. Therefore, we reasoned to explore the involvement of RTKs into the definition of PDAC subtypes which display distinct biological and clinical behaviours. The FGF-FGFR axis has been reportedly involved in pancreatic cell specification [22–25] and, while an aberrant FGFR signalling is reported in several malignancies [46, 47], its involvement in PDAC has been largely neglected so far. Amplification and overexpression of FGFR1 are described in up to 10% of patients with advanced PDAC [48] but rarer in resectable cohorts [3, 4]. Expression of FGFR4 is reported in a substantial fraction of PDAC but its role in mediating tumorigenesis and therapy resistance has been seldomly explored [49, 50]. Here, we provide multiple lines of evidence that *FGFR4* expression is restricted to epithelial cells in both normal and diseased pancreata, selectively elevated in classical tumours, and correlated with better outcomes. As opposed to *FGFR4*, *FGFR1* showed more promiscuous expression in both normal and diseased pancreata, was strongly associated with the EMT phenotype but not with epithelial (i.e. classical or basal-like) PDAC subtypes. Accordingly, genetic manipulation of *FGFR1* in cells displaying mesenchymal traits and classified as basal-like based on transcriptomic profiling resulted in a reduced expression of markers/drivers of EMT and of the EMT gene program but did not lead to epithelial phenotype switch. Increased *FGFR1* expression could be observed in PDAC cells following prolonged exposure to TGFβ1 and coherently reduced upon transient knockdown of the master-regulator ZEB1. Activation of FGFR1 is linked to EMT in prostate cancer [51], and both EMT and upregulation of FGFR1 has been linked to resistance to targeted therapies in lung cancers [52, 53]. In our study, selective inhibition of FGFR1 with BGJ398 did not dramatically affect proliferation of PDAC cells. Differently from FGFR1, we found a defined relationship between *FGFR4* expression and the classical subtype of PDAC. Loss of *FGFR4* in basal-like/squamous tumours was concordant with hypermethylation of the gene and reduced levels of active chromatin in its regulatory regions. Our results strongly suggest an epigenetic dysregulation of *FGFR4* expression in basal-like/squamous tumours which is similar to that observed for several endodermal transcription factors. We also demonstrated that *FGFR4* is a direct target of the classical transcription factor HNF1A. In line with recent reports, the silencing of *HNF1A* in the classical PDAC cell line HPAF-II was not sufficient to drive a subtype switch. In the same model, however, downregulation of *FGFR4* was sufficient to drive a full subtype switch thus suggesting that FGFR4 has stronger “antibasal” function than HNF1A in this cell line. In other two well-established models of classical/progenitor PDAC, the forced downregulation of *FGFR4* was not sufficient to drive a full switch of phenotype towards the basal-like subtype but was invariably associated with the reduced expression of classical genes and the enrichment for transcriptional programs of basal-like/squamous PDAC. This suggests that FGFR4 acts to maintain the classical phenotype and therefore to limit the expression of ectopic gene programs (i.e. basal-like/squamous) in PDAC. In keeping with that, the analysis of scRNA-Seq data demonstrated that, at individual cell level, FGFR4 expression is elevated in cells with a more classical/epithelial phenotype.

We additionally showed that FGFR4 loss is associated with enhanced malignancy of PDAC cells by increasing in vitro and in vivo proliferation as well as enhancing metastatization of a cell line of the classical background. This finding further corroborates the evidence that basal-like cells tend to accumulate in advanced stages of the disease. While repression of “basalness/squamousness” seems an intrinsic feature of FGFR4, we also showed that its silencing in *FGFR4*^*low*^ basal-like models increases malignant behaviour suggesting a bona fide tumour suppressive role for endogenous levels of FGFR4 in PDAC. Others have recently suggested that RTKs in PDAC might exert an oncogenic role only when overexpressed [54]. In keeping with this, acceleration of cell proliferation could be observed in established and primary cell lines regardless of their background and upon either transient or stable knockdown of *FGFR4*. Accordingly, gene programs related to cell cycle and proliferation were highly enriched in the comparison between low versus high *FGFR4* tumours of the ICGC and PanCuRx cohorts [3, 5]. Pharmacological inhibition of FGFR4 had little to no effect on the short-term proliferation of several PDAC cell lines. Interestingly, stimulation of *FGFR4*^*high*^ classical cells with FGF2 and FGF19 led to reduced cell proliferation, which partially agrees with results from Motoda et al. [50] that suggested a tumour suppressive role of FGF19 stimulation on cells expressing FGFR4 in PDAC. Conversely, FGF ligands did not exert inhibitor effects on the proliferation of cells deficient for FGFR4. Mechanistically, downregulation of *FGFR4* was associated with increased basal fluxes through the MAPK and the mTORC1 signaling pathways regardless of the genomic and transcriptomic background of the cells. Accordingly, PI3K/Akt/mTOR transcriptional signatures as well as the inhibitory phosphorylation of one of the best characterised mTORC1 substrate, 4E-BP1, were significantly enriched in both cell lines and tissues displaying reduced expression of FGFR4. Phosphorylation of 4E-BP1 by mTORC1 has been demonstrated to regulate mTORC1-driven proliferation in mammalian cells [44, 45] and accordingly its levels were higher in all cell lines tested following silencing of *FGFR4* and were concordant with increased cell proliferation and protein synthesis. In keeping with this, pharmacological inhibition of mTORC1 with Everolimus showed superior effect in reducing the proliferation of cells deficient for *FGFR4* as opposed to proficient cells. In summary, we show that endogenous levels of *FGFR4* limit the malignant phenotype of PDAC. In particular, the loss of FGFR4 was associated with increased activity of the mTORC1 pathway in PDAC cells. Finally, we propose *FGFR4* as a valuable marker for the prognostic stratification of PDAC patients.

## Supplementary information

Supplemental Material

Supplemental Figure 1

Supplemental Figure 2

Supplemental Figure 3

Supplemental Figure 4

Supplemental Figure 5

Supplemental Figure 6

Supplementary Tables

## Data Availability

The primary RNA-Seq data generated for this study will be available in a public, open access repository. All other relevant data are already available as Supplementary material.
