# Supplementary material for: Loss of FGFR4 promotes the malignant phenotype of PDAC
Source: Oncogene. 2022 Aug 13;41(38):4371–84. doi: 10.1038/s41388-022-02432-5 (PMC9481460; doi:10.1038/s41388-022-02432-5)
Supplement: Supplementary file 1 — Supplemental Material [file 41388_2022_2432_MOESM1_ESM.docx]

**ONLINE SUPPLEMENTAL MATERIAL**

**Index**

- Supplemental tables description
- Supplemental Figure Legends
- Materials and Methods
- References to Supplemental Material

**SUPPLEMENTAL TABLES DESCRIPTION**

**Table S1.** Clinicopathological data of the resected PDAC cases.

**Table S2**. List of differentially expressed genes upon FGFR1 knockdown in Hs766T cell line; Related to Supplemental Figure 3~~.~~

**Table S3**. Gene-set enrichment analysis (GSEA) on the list of differentially expressed genes upon FGFR1 knockdown in Hs766T cell line; Related to Figure 2 Supplemental Figure S3. (A) GSEA analysis of the up- and downregulated genes in BioCarta gene sets. (B) GSEA analysis of the up- and downregulated genes in GO gene sets. (C) GSEA analysis of the up- and downregulated genes in Hallmark gene sets. (D) GSEA analysis of the up- and downregulated genes in KEGG gene sets. (E) GSEA analysis of the up- and downregulated genes in Reactome gene sets

**Table S4**. List of differentially expressed genes following knockdown of FGFR4 in HPAF-II cell line; Related to Figure 3 and Supplemental Figure 4.

**Table S5**. Gene-set enrichment analysis (GSEA) on the list of differentially expressed genes upon FGFR4 knockdown in HPAF-II cell line; Related to Figure 3. (A) GSEA analysis of the up- and downregulated genes in BioCarta gene sets. (B) GSEA analysis of the up- and downregulated genes in GO gene sets. (C) GSEA analysis of the up- and downregulated genes in Hallmark gene sets. (D) GSEA analysis of the up- and downregulated genes in KEGG gene sets. (E) GSEA analysis of the up- and downregulated genes in Reactome gene sets.

**Table S6**. List of differentially expressed genes following knockdown of FGFR4 in HPAF-II cell line; Related to Figure 5.

**Table S7**. Gene-set enrichment analysis (GSEA) on the list of differentially expressed genes upon FGFR4 knockdown in HPAF-II cell line; Related to Figure 5. (A) GSEA analysis of the up- and downregulated genes in BioCarta gene sets. (B) GSEA analysis of the up- and downregulated genes in GO gene sets. (C) GSEA analysis of the up- and downregulated genes in Hallmark gene sets. (D) GSEA analysis of the up- and downregulated genes in KEGG gene sets. (E) GSEA analysis of the up- and downregulated genes in Reactome gene sets.

**Table S8.** Gene-set enrichment analysis performed with the tool enrichR on the list of differentially expressed genes for three different datasets. (A) FGFR4 knockdown in HPAF-II cell line. (B) FGFR4^low^ vs FGFR^high^ tumours in the ICGC cohort. (C) FGFR4^low^ vs FGFR^high^ tumours in the PanCuRx cohort. Terms highlighted in light blue are represented in the corresponding Fig. 5A and 5B; terms highlighted in yellow are represented in Supplemental Figure 6H.

**SUPPLEMENTAL FIGURE LEGENDS**

**Supplementary Figure 1. Expression of *FGFR4* and *FGFR1* in pancreatic samples (A)** Violin plots of the normalized expression of FGFR4 (left panel) and FGFR1 (right panel) in each annotated cell cluster from the scRNA-seq of normal adult pancreas from 3 different datasets[1-3]. (**B**) Representative ISH images showing expression of *FGFR4* and *FGFR1* in normal pancreas. Scale Bar, 100 µm. Insets show magnification of selected areas: 1, islet of Langerhans; 2, acinar cells; and 3, ductal cells. (**C**) Density plot showing the distribution the *FGFR4* and *FGFR1* mRNA expression levels in the PanCuRx cohort [4]. The dashed vertical lines refer to the value of mRNA levels (vst) that has been used as threshold to define low- versus high-expressing samples. (**D**) GSVA score (using ssgsea method) for the squamous signature[5] for each sample of the PanCuRx cohort [4] according to the expression of *FGFR1* and *FGFR4.* *, p < 0.05; **, p < 0.01; ****, p < 0.0001; and ns, not significant as determined by Wilcoxon test. (**E**) Stacked bar plot showing the Moffitt molecular subtypes distribution according to the *FGFR4* expression status in the ICGC (left) and the PanCuRx cohorts (right). (**F**) Stacked bar plot showing the Moffitt molecular subtypes distribution according to the *GATA6* expression status in the ICGC (left) and PanCuRx (right) cohorts. (**G**) Stacked bar plot showing the molecular subtypes distribution according to the *FGFR4* expression status in the PanCuRx cohorts.

**Supplementary Figure 2. Expression of *FGFR4* and *FGFR1* in tissues and *in vitro* models of PDAC**. (**A**) Representative ISH images of PDAC tissues showing no (0), moderate (1) or high (2) expression of *FGFR4* or *FGFR1*. Scale bars, 60 µm. (**B**) Stacked bar plot showing the distribution of morphological grades according to the *FGFR4* expression status in the PDAC tissue cohort. **, p < 0.01 by two-sided Chi-square test. **(C**) Representative ISH images of PDAC tissues showing either poor (top) or well (bottom) differentiation; scale bar, 60 µm. (**D**) Immunoblot analysis for ZEB1, ErbB3, E-Cadherin, and Vimentin in whole-cell lysates of 6 PDAC cell cultures. β-Actin was used as loading control. (**E**) Representative immunofluorescence staining of PDX1 (green) and CK5 (red) in four PDAC cell lines. Counterstain, DAPI (blue); magnification 100x. Quantification of PDX1+ cells and CK5+ cells in four PDAC cell lines is provided in the right panel as number of positive cells in a minimum of 5 field of visualization (FOV) per cell line. (**F**) Representative fluorescence *in situ* hybridization of *FGFR4* and *FGFR1* in 6 PDAC cell cultures. DAPI, blue; *FGFR1*, green; *FGFR4*, red. **(G)** Immunoblot analysis of FGFR1 and FGFR4 in whole-cell lysates of 5 PDAC organoid cultures. Vinculin was used as loading control. The classical and the basal-like signature scores were calculated for each PDAC organoid cultures using GSVA (ssgsea method).

**Supplementary Figure 3. FGFR1 is associated with the EMT phenotype in PDAC. (A)** cBioportal oncoprint visualization of genetic alterations (single-nucleotide variations, copy-number variations, and structural variations) in samples from the ICGC[5] and TCGA[6] cohorts. (**B-C**) cBioportal Boxplot visualization of the correlation between *FGFR1* (B) and *FGFR4* (C) mRNA and copy-number status for samples of the ICGC[5] and TCGA[6] cohorts. **(D)** Classification of primary PDAC cell cultures based on RNA-Seq. At the bottom, immunoblot of whole-cell lysates showing different levels of FGFR4. Vinculin, loading control. **(E)** Competition based-assay in PD9-O following infection with the shRNA targeting FGFR1. Representative phase-contrast images (top), GFP signal (green, middle panel) and merged images (bottom panel) demonstrating loss of GFP positivity over time. Scale bar as indicated. At the bottom, quantification of the GFP positivity (as percentage of signal compared to P0) over time of PDA9-0 transduced with the indicated vector linked to GFP. Data presented as mean + SEM (n = 3). **(F)** Volcano plot of differences in gene expression between control (n = 3) and *FGFR1* knocked down (KD) cells (n = 3). Indicated are some of the genes with log_2_FC expression ≥ 2 and adjusted *p* < 0.05. See also Supplemental Table 2. (**G**) Enrichment of pathways in Hs766T following *FGFR1* knock-down. The GSEA analysis was performed using gene sets from HALLMARK database in MsigDB library. Displayed are gene sets that passed FDR < 0.05. See also Supplemental Table 3.

**Supplementary Figure 4**. **Loss of FGFR4 enhances the malignancy of PDAC cells.** **(A)** Representative snapshot of the genomic region of *FGFR4* in MiaPaCa2 (high-grade, basal-like) and Capan-1 (low-grade, classical) from Diaferia et al.[7] showing histone modification (H3K27ac) and RNA-seq data. (**B**) Volcano plot of differences in gene expression between control (n = 2) and *HNF1A* knocked down (KD) cells (n = 3). Indicated are some of the genes with log_2_FC expression ≥ 2 and adjusted *p* < 0.05. See Supplemental Table 4 for the full list of differentially-expressed genes. (**C**) Stacked bar plot showing the distribution of primary and metastatic cases from the PanCuRx cohort according to the FGFR4 expression status. p, Chi-Square test. (**D**) Immunoblot for FGFR4 in hT3 PDO transduced with either mock control of the shRNA targeting FGFR4. Vinculin was used as loading control. The transplantations were performed with the hT3 transduced with the sh884. (**E**) Representative ISH staining of *FGFR1* in PDA9-O transduced with NTC or shFGFR4. Scale bar, 50 μm. (**F**) Relative growth (as percentage of cell proliferation) of HPAF-II, PDA23, PDA9, and PDA2 cells transiently transfected with either the control vector (NTC) or the siRNA targeting *FGFR4*. Data presented are means ± SD of 3 biological replicates. (**G**) Enrichment of selected pathways (GSEA) when comparing *FGFR4* low versus *FGFR4* high tumours of the ICGC cohort[5]. GSEA was performed using gene sets from REACTOME, GO, Hallmark, and REACTOME databases in MsigDB library. Displayed gene sets that passed false-discovery rate < 0.05.

**Supplementary Figure 5. Transcriptional changes associated with the downregulation of FGFR4 in pancreatic cancer.** (**A**) Expression of classical and basal-like genes in *FGFR4* low versus *FGFR4* high tumours of the ICGC[5] cohort. ****, false discovery rate (fdr) < 0.0001; ***, fdr < 0.001; **, fdr < 0.01; and *, fdr < 0.1 by Wald test. (**B**) Enrichment of Interferon related pathways when comparing *FGFR4* low versus *FGFR4* high tumours of the ICGC[5] cohort. (**C**) Immunoblot analysis of FGFR4 in whole-cell lysates of 3 PDAC cultures transfected with either mock control or siRNA targeting FGFR4. Vinculin was used as loading control. The progenitor and the squamous signature scores were calculated for each culture using GSVA (ssgsea method) and are based on the classification proposed by Somerville et al.[8]. (**D**) GSEA plot evaluating the Gene Program 5 (GP5, c-Myc activity) from the Bailey classificator[5] upon *FGFR4* knockdown in AsPC1 cell line. (**E**) Enrichment of Myc-related and proliferation-related pathways upon downregulation of *FGFR4* in AsPC1. (**F-G**) Expression of classical (blue) and basal-like (red) markers in cells depleted of *FGFR4* (as compared to parental cells). (**H**) Immunoblot analysis of E-cadherin in SUIT2 and AsPC1 cells upon knockdown of FGFR4. GAPDH was used as loading control. (**I**) Expression of *FGFR4* in ductal cells classified as classical or basal-like from Peng et al.[9]. ****, p < 0.01 by Student t test. (**J**) Samples from Peng et al. ranked by mean *FGFR4* expression in the epithelial compartment. The cases in blue and red were considered as *FGFR4* low (n = 4) and high (n = 4), respectively. (**K**) Expression of classical/progenitor and basal-like/squamous markers in *FGFR4^high^* and *FGFR4^low^* epithelial cells from J.

**Supplementary Figure 6. Loss of FGFR4 is associated to hyperactivation of mTORC1 in PDAC. (A)** Immunoblot analyses of p-FRS2, p-ERK1/2 in whole cell lysates from HPAF-II transfected with either control (NTC) or siRNA targeting *FGFR4.* GAPDH was used as loading control. Cells were serum-starved for 8 hours and then either left untreated or stimulated with FGF2 (25 ng/mL), and 2% CS-FBS for 20 minutes. (**B**) Immunoblot analyses of p-ERK1/2, total ERK1/2 in whole cell lysates from SUIT2 transfected with either control (NTC) or siRNA targeting *FGFR4.* Vinculin was used as loading control. Cells were serum-starved for 8 hours and then either left untreated or stimulated with FGF2 (25 ng/mL), FGF19 (100 ng/mL), and 2% CS-FBS for 20 minutes. (**C**) Immunoblot analyses of p-STAT3, and total STAT3 in whole cell lysates from HPAF-II, and SUIT2 cell lines (in blue). For SUIT2, the same cell lysates from B were used, therefore the same Vinculin blot is used for normalization. Bottom panel, immunoblot analysis of p-STAT3, total STAT3, p-ERK1/2, and total ERK1/2 for AsPC1 (in red). Vinculin was used as loading control. Numbers on top of the p-STAT3 and p-ERK1/2 refers to the quantification of the phosphorylated levels by normalizing first for the loading control and then for total level of the proteins. For all panels, cells were serum starved and treated as indicated. **(D)** Bar plots showing the quantification of changes in the phosphorylated levels of selected proteins (p-AKT, p-S6, and p-4E-BP1) as relative density of the total protein level. From Fig 6C. Data presented as means ± SD of four biological replicates. *, *p* < 0.05; **, *p* < 0.01; ***, *p* < 0.001 by Student’s *t-*test. **(E-G)** Immunoblot analyses of FGFR4, and selected components of the mTORC1 pathway in whole cell lysates from 3 different primary PDAC cell lines transfected with either control (NTC) or siRNA targeting *FGFR4*. GAPDH was used as loading control. Cells were serum-starved for 8 hours and then either left untreated or stimulated with FGF2 (25 ng/mL), FGF19 (100 ng/mL), and 2% CS-FBS for 20 minutes. Quantification of changes in the phosphorylated levels of selected proteins (p-S6, and p-4E-BP1) is provided in the bar plots on the right. (**H**) Immunoblot analyses of p-4E-BP1 in whole cell lysates from AsPC1 and SUIT2 cell lines treated as in E-G. For both AsPC1 and SUIT2, the same cell lysates from B were used, therefore the same Vinculin blot is used for normalization using the serum-starved mock-transfected cell lines as reference. (**I**) EnrichR pathway analysis of significantly over-represented genes in HPAF-II lacking FGFR4. See also Supplemental Table 8.

**MATERIALS AND METHODS**

**Human samples**

Pancreatic cancer tissues used in this study were obtained from surgical resections of patients treated at the University and Hospital Trust of Verona (Azienda Ospedaliera Universitaria Integrata, AOUI). Written informed consent from the donors for research use of the tissue was obtained prior to acquisition of the specimens. In particular, the FFPE tissues of 106 PDAC cases used for *in situ* hybridization and immunohistochemical analyses were retrieved from the ARC-Net Biobank and were collected under the protocol number 1885 approved by the local Ethics Committee (*Comitato Etico Azienda Ospedaliera Universitaria Integrata*) to A.S. (Prot. 52070, Prog. 1885 on 17/11/2010). Two different pathologists (A.S. and C.L.) independently performed histopathological evaluation and scoring of the tissues for *FGFR1* and *FGFR4* expression. Resected tissues used for the generation of organoids and monolayer cell cultures were collected under the protocol number 1911 approved by the local Ethics Committee (*Comitato Etico Azienda Ospedaliera Universitaria Integrata*) to V.C. (Prot. n 61413, Prog 1911 on 19/09/2018). All experiments were conducted in accordance with relevant guidelines and regulations.

**Cell cultures**

A total of 12 monolayer cell cultures and 5 PDAC organoids were used in this study. The established human cell lines HPAF-II, PANC1, and AsPC-1 were purchased from ATCC (CRL-1997™, CRL-1469™, CRL-1682^TM^). Human hM1, hF2, and hT1 2D cell cultures and hT3 organoids were kindly provided by Dr. David Tuveson (Cold Spring Harbor Laboratory, NY, USA), while SUIT-2 and Hs766T were kindly provided by Prof. Aldo Scarpa (University of Verona). HPAF-II, PANC1, SUIT-2, and AsPC-1 were grown in DMEM (Gibco) supplemented with 10% FBS and 1% Penicillin-Streptomycin (Pen-Strep, Gibco). hM1, hF2, Hs766T, and hT1 were cultured in RPMI (Gibco) supplemented with 10% FBS and 1% Pen-Strep. PDAC organoids and primary monolayer cultures were established as described previously [10, 11]. Briefly, tumour specimens were minced and digested with Collagenase II (5 mg/mL, Gibco) and Dispase I (1.25 mg/mL, Gibco) in human complete medium (described in [10]) at 37°C for a maximum of 2 hours. The resulting material was further digested with TrypLE (Gibco) for 10 minutes at 37°C, embedded in growth factor reduced Matrigel^(R)^ and cultured in human complete medium. Of the models used here, 7 out of 12 carry the G388R variant of FGFR4 (HPAF-II, hF2, SUIT2, PDA23, PDA9, PANC1, and hT3).

Four of the five organoid models used were acquired as part of the Human Cancer Model Initiative (HCMI) <https://ocg.cancer.gov/programs/HCMI> and, those models are available for access from ATCC. The corresponding IDs are as follows:

| **Sample Code** | **ID** |
| --- | --- |
| PDA1-O | HCM-CSHL-0080-C25 |
| PDA2-O | HCM-CSHL-0077-C25 |
| PDA9-O | HCM-CSHL-0081-C25 |
| PDA20-O | HCM-CSHL-0092-C25 |

Tissue digestion from 4 specimens was also directly seeded on tissue-culture vessels for initiation of monolayer cell cultures using the following medium: Advanced DMEM/F12 medium (Gibco) supplemented with HEPES (1X, Gibco), Glutamax^TM^ (1X, Gibco), Primocin^TM^ (1 mg/mL, Invivogen), mouse Epidermal Growth Factor (50 ng/mL, Gibco), Dexamethasone (3 nM, Sigma), and 5% Fetal Bovine Serum (FBS, Gibco). Both monolayer cell cultures and organoids were routinely tested for the presence of mycoplasma using MycoAlert Detection Kit from Lonza, in accordance with the manufacturer’s instructions.

**Lentiviral infection of cell lines and organoids**

Stable knockdown of *FGFR1* and *FGFR4* was achieved using validated short hairpin RNAs (shRNA) against human *FGFR1* (TRC clone 574) and human *FGFR4* (TRC clone 884, 885, and 419) in the vector pLKO.1-puro-CMV-tGFP (Sigma-Aldrich). The vector TRC2 pLKO.5-puro (Sigma-Aldrich) was used as non-targeting control (NTC) of the infection. Lentiviruses were produced in HEK293T cells by transfecting plasmids (containing the GFP cassette) and the packaging plasmid VSV-G with X-tremeGENE9 (Roche, 063665110101). The viral supernatants were harvested at 48 hours and cell debris removed prior to infection. To concentrate lentivirus, Lenti-X concentrator (TaKaRa Bio) was used. Transduction of monolayer cell cultures was performed by adding the viral supernatants supplemented with 1 µg/mL of Polybrene (Santa Cruz Biotechnology) to cells having 50-60% of confluency. Two days after infection, 2 µg/mL of puromycin (Gibco) was added for antibiotic selection. For transduction of organoids, cultures were first released from Matrigel^(R)^  by incubation with a solution of Dispase I at 37°C for 20 minutes, and then subjected to enzymatic digestion with TripLE (Gibco) supplemented with Dispase I and 0.1 mg/mL DNAse I (Sigma-Aldrich) for 20 minutes. 1x10^5^ single cells were resuspended with the transduction medium supplemented with 1 µg/mL of polybrene, spinoculated at 600 x g for 1 hour at room temperature, and incubated for 16 hours at 37°C. The following day, cell suspension was recovered and seeded in Matrigel^(R)^. Two days after infection, cells were treated with 2 µg/mL puromycin (Gibco) for antibiotic selection. Successful transduction was assessed by visualizing GFP positivity of cells with the EVOS Cell Imaging System (Thermo Fisher Scientific), and antibiotic selection stopped when the treatment caused extinction of the untransduced cultures. The sequences of shRNAs against *FGFR1* and *FGFR4* are as follows:

| **Target location** | **Clone ID** | **DNA Sequence** |
| --- | --- | --- |
| 3UTR | TRCN0000199419 | CCTGACACAGTGCTCGACCTT |
| CDS | TRCN0000219885 | TGTGGGCAGCATCCGCTATAA |
| CDS | TRCN0000219884 | CCCTCGAATAGGCACAGTTAC |
| CDS | TRCN0000312574 | TGCCACCTGGAGCATCATAAT |

For in vitro drop-out experiments with organoids, the viral supernatants were adjusted to achieve an 80% of GFP+ ratio. As control, organoids were transduced with pre-made GFP expressing lentiviral particles (CMV-neo, Amsbio).

**Animal Studies**

Six- to Eight-weeks old *NSG* (NOD.Cg-Prkdc^scid^;Il2rg^tm1Wjl^) mice were purchased from Charles River Laboratory (Milan). All animal experiments regarding transplanted mice were conducted in accordance with procedures approved by CIRSAL at University of Verona (approved project 655/2017-PR). No statistical methods were used to predetermine sample size estimate for transplantation experiments, which was instead based on preliminary experiments showing an engraftment rate above 95% for both cell lines and organoids. Therefore, a minimum of 5 successful grafts (per group) was considered suitable to consistently identify differences between groups in terms of tumour growth. For orthotopic transplantation of either cell lines (1x10^6^ cells) or dissociated organoids (1x10^6^ cells), mice were anesthetized using isoflurane and an incision was made in the left abdominal side at the level of the spleen. Cells were resuspended in 50 µl of a 2:3 dilution of Matrigel^(R)^  (Corning) and cold PBS (Gibco) and injected into the tail region of the pancreas using insulin syringes (BD micro-fine 30 Gauge). The injection was considered successful by the development of bubble without signs of leakage. Mice with signs of leakage upon injection were excluded from the studies. The abdominal wall was sutured with absorbable vicryl sutures (Vetsuture), and the skin was closed with wound clips (CellPoint Scientific Inc.). Monitoring of tumour growth was performed as previously described [12]. Briefly, following weekly manual palpation starting 10 days following transplantation, tumour-bearing mice were subjected to high-contrast ultrasound screening using the Vevo 2100 System with a MS250, 13–24 MHz scanhead (Visual Sonics). Mice were sacrificed at the indicated time points (4-5 weeks, once the tumour masses have reached the 10 mm in diameter). Pancreas, spleen, lungs and liver were collected for downstream analysis.

**Transient Knockdown in Cell lines**

For small interfering RNA (siRNA) knockdown experiments, siRNA constructs were purchased from Thermofisher Scientific (s5176, s229971, s13869, s5164) and primary cell cultures or established cell lines were transfected with either 25 or 50 pmol of siRNA using Lipofectamine^TM^ 2000 (Thermofisher Scientific, #11668027) according to manufacturer’s instruction for 6-well plates format. The sense strand of the duplex siRNAs was: *FGFR4*, 5' CAUUGACUACUAUAAGAAAtt 3’; *ZEB1*, 5’GGUAGAUGGUAAUGUAAUATT 3’; *HNF1A*, 5’ AGACUAUGCUCAUCACCGATT 3’; and *FGFR1*, 5’ GAGGCUACAAGGUCCGUUAtt 3’. Non-targeting siRNA (Ambion) was used as negative control at a final concentration of 25 pmol.

**Cell Proliferation Assay**

For measuring cell proliferation, 2x10^3^ cells were plated on white 96-well plate (Thermofisher Scientific) and cultured in 100 μL of culture medium. Cell viability was measured 72 hours post-seeding using the CellTiter-Glo assay (Promega, G9683).

**Fluorescent activated cell sorting**

Fluorescent activated cell sorting (FACS) analysis was performed in order to evaluate the percentage of GFP positive cells. Before the analysis, organoids were first released from Matrigel^(R)^  by incubation with a solution of Dispase I at 37 °C for 20 minutes, and then subjected to enzymatic digestion with TripLE supplemented with Dispase I and 0.1 mg/mL DNAse I (Sigma-Aldrich) for 20 minutes, until the organoids appeared as single cells under the microscope. Cells were filtrated (Cell Strainer 40μm Nylon, BD Falcon), resuspended in cold PBS and acquired using FACS Canto II (BD Biosciences). Data were analysed using FlowJo software (Flowjo LLc). Untransduced and GFP-stably expressing cells were used as negative and positive controls, respectively.

**Nascent protein detection**

To visualize nascent proteins, the Click-iT Protein Synthesis Assay Kit reagents (Invitrogen) was used. Cells were plated on black 96-well plates (Thermofisher Scientific) and cultured for 24 hours in DMEM methionine-free (Gibco) medium supplemented with 1X HEPES and 1X Glutamax. Cells were transfected with siRNA as previously described. Two days post-transfection, cells were incubated with the methionine analog L-homopropargylglycine (HPG) for 30 minutes at final concentration of 50 µM in methionine-free medium under conditions optimal for the cells (humidified atmosphere at 37°C and 5% CO2). After washing with cold PBS, cells were fixed with 3.7% formaldehyde (Sigma-Aldrich) for 15 minutes at room temperature, followed by a permeabilization step using 0.5% Triton X-100 (Sigma-Aldrich). The Click-iT reaction, with Alexa-488 detection reagents, was performed according to the manufacturer’s protocol. For the control experiment and to confirm the specificity of the signal, 300 µg/mL cycloheximide (Sigma-Aldrich), a protein synthesis inhibitor, was added 30 minutes before HPG addition. After the Click-iT reaction, cell plates were scan using VICTOR 3 1420 multilabel counter plate reader (PerkinElmer) to assess nascent protein synthesis by determining signal intensity in the fluorescent channel of FITC. Two dimensional cultures were also imaged using Axio Observed Z1/7 inverted Wide field microscope (Zeiss) with 40x objective magnification and digitalized by the Zeiss Zen software.

**Therapeutic experiments with cell cultures**

BGJ398 (S2183, Selleckchem), BLU3391 (S7819, Selleckchem), and Everolimus (S1120, Selleckchem) were dissolved in DMSO (Sigma-Aldrich). The final concentration of DMSO was no higher than 0.2%. Briefly, cells were dissociated to single cells by enzymatic dissociation with 1X Trypsin (Gibco) at 37°C for 2 minutes, counted and seeded in a range concentration of 1x10^3^ – 2.5x10^3^ cells into individual wells of a white 96-well plate. After 24 hours, cells were treated with appropriate drugs for 48 hours using drug concentrations ranging from 0.1 nM to 10 µM. Proliferation was measured using CellTiter-Glo assay per the manufacturer’s protocol.

For treatment with Everolimus, cells were counted and diluted to obtain 20 cells/µL in 100 µL of Opti-MEM^TM^ supplemented with 5% FBS. 100 µL of cells-containing medium was plated into individual wells of a white 96-well plate. Twenty-four hours after culture, cells were transfected with siRNA against FGFR4 and non-targeting small interfering RNA control, as previously described. The concentration of Everolimus necessary to achieve inhibition of activity by 50% (IC_50_) was calculated using concentrations ranging from 0.1 nM to 10 µM (7-point curve). 1 µM of drug was added in 100 µL of culture medium 48 hours post-plating, with nine replicate wells for each sample. At day 5, cell viability was measured using a luminescence ATP-based assay (CellTiter-Glo). Cells were also treated with 5 ng/mL of human TGFβ1 (#100-21, Peprotech), alone or in combination with 500 nM of A83-01 (#2939, Tocris Bioscience), 25 ng/mL of Recombinant Human FGF-basic (#100-18C, Peprotech), 100 ng/mL of Recombinant Human FGF10 (#100-26, Peprotech), 100 ng/mL of Recombinant Human FGF19 (#100-32, Peprotech), and 2% of Fetal Bovine Serum (FBS, Gibco).

**Histology and Immunohistochemistry**

Tissues were fixed in 10% neutral buffered formalin and embedded in paraffin. Sections were subjected to Hematoxylin and Eosin as well as immunohistochemical staining. The following primary antibody was used for immunohistochemical staining of xenografts: Phospho-4E-BP1 (Thr37/46) (#2855, Cell Signaling), 1:400. Slides were scanned at 40x magnification and digitalized using the Aperio Scan-Scope XT Slide Scanner (Aperio Technologies). Quantification of p-4E-BP1 staining was performed in at least five random nonoverlapping fields of visualization (magnification, 20x) in each sample using ImageJ. To measure the percentage of positive area, captured images were first color deconvoluted and DAB+ particles counted automatically. The percentage of strong positive pixels was calculated relative to the number of nuclei present in each of the selected areas. Positivity for p-4E-BP1 was rarely seen in non-neoplastic cells.

**Immunofluorescence**

Cells (5 x 10^3^ cells per chamber) were seeded into chamber culture slides (BD Falcon) in culture media. The next day, cells were rinsed with ice-cold PBS and fixed with 3.7% formaldehyde for 10 minutes at room temperature followed by permeabilization with 0.5% Triton X-100. The cells were subjected to immunofluorescence staining with the following primary antibodies: PDX1 (#EPR3358, ab134150, Abcam), 1:100; and Cytokeratin 5 (CK5) (NCL-L-CK5, Leica Biosystems), 1:100. All antibodies were incubated for 2 hours at room temperature. The cells were then washed with cold PBS three times for 3 minutes and incubated with Alexa 488-labeled anti-rabbit secondary antibody (1:500) (Invitrogen), and Alexa 555-labeled anti-mouse secondary antibody (1:500) (Invitrogen) at room temperature for 1 hour. A final step of washes in cold PBS was completed at room temperature followed by mounting with DAPI (Vector Laboratories). The cells were examined by Olympus BX61 Upright fluorescence microscope (Leica).

***In Situ* Hybridization**

The *in-situ* hybridization (ISH) was performed on 4 μm section of human PDAC tissues (n = 106) and on xenografts. Briefly, sections were deparaffinized by incubation with xylene for 10 minutes, 100% ethanol for 2 minutes and then let dry for 5 minutes at room temperature. Slides were incubated for 10 minutes with RNAscope® Hydrogen Peroxide (Advanced Cell Diagnostics), washed with distilled water and incubated for 20 minutes at 99°C with RNAscope® 1X Retrieval Reagents (Advanced Cell Diagnostics). Sections were rinsed in distilled water and dehydrated in 100% ethanol for 3 minutes and let dry at room temperature. Then, the slides were incubated at 40°C for 10 minutes with RNAscope® Protease Plus (Advanced Cell Diagnostics), washed with distilled water and incubated with the appropriate probe for 2 hours at 40°C, followed by washes with RNAscope® 1X Wash Buffer (Advanced Cell Diagnostic). The different RNAscope® 2.5 HD AMPs (Hs-FGFR1-C2 and Hs-FGFR4-no-XMm-C1, Advanced Cell Diagnostics) were added to the slides following manufacturer’s instructions. Positive control probe Hs-UBC and 2-plex negative control probe (Advanced Cell Diagnostics) were used as positive and negative control, respectively. Then, slides were incubated for 10 minutes at room temperature with either ImmPACT™ DAB Substrate Kit (DBA) or RNAscope® 2.5 HD Detection Reagent- RED (Advanced Cell Diagnostic). Slides were stained with hematoxylin, dried at 60°C for 15 minutes and mounted with VectaMount® mounting medium (Vector Laboratories). Slides were scanned at 40x magnification and digitalized using the Aperio Scan-Scope XT Slide Scanner. For each case, two consecutive tissue sections were prepared and stained for *FGFR1* and *FGFR4*. Only RNA signals from neoplastic cells were evaluated in a minimum of 5 field of investigations (at 20X magnification). The following semiquantitative method was used for scoring: score 0, no or rare signals (dots); score 1, between 3 and 10 dots per cell at 20X magnification; score 2, more than 10 dots (or clusters) per cell. For each marker, cases were considered high if showing an average score > 1.5, and low if showing a score of < 1.5. A total of 97 cases were suitable for evaluation of the mRNA expression of both *FGFR1* and *FGFR4*; of those 76 had available overall survival data for correlation analyses.

**Immunobloting**

Protein lysates were prepared using Cell Signaling Lysis Buffer (Cell Signaling) and separated on 4-12% Bis-Tris NuPAGE gels (Life Technologies), transferred onto a PVDF membrane (Millipore) and incubated with the following antibodies: FGFR4 (8562, Cell Signalling), FGFR1 (9740, Cell Signalling), Akt (9272, Cell Signalling), phospho-Akt (4060, Cell Signalling), phospho-S6 Ribosomal protein (4858, Cell Signalling), S6 Ribosomal protein (2217 ,Cell Signalling), phospho-4E-BP1 (2855, Cell Signalling), 4E-BP1 (9644, Cell Signalling), ERK1/2 (9102, Cell Signalling), phospho-ERK1/2 (4376, Cell Signalling), phospho-FRS2 (2864, Cell Signalling), STAT3 (12640, Cell Signalling), phospho-STAT3 (Tyr705, 9145 Cell Signalling), Vimentin (NCL-L-VIM-V9, Leica Biosystems), HER3/ErbB3 (12708, Cell Signalling), ZEB1 (203829, Abcam), HNF1A (sc-393925, Santa Cruz Biotechnology), E-Cadherin (M3612, Dako), RAS (F234, Santa Cruz Biotechnology). Vinculin (4650, Cell Signalling), GADPH (5174, Cell Signalling) (sc-166545, Santa Cruz Biotechnology), β-Actin (4967, Cell Signalling) were used as loading control. The immunoblots were visualized with ECL plus (Amersham/GE Healthcare Europe GmgH).

**qRT-PCR analysis**

RNA was extracted with Trizol® Reagent (Life Technologies) method. 1 µg of DNase-treated RNA was reverse transcribed using TaqMan® Reverse Transcription reagents (Applied Biosystems) in a volume of 20 µL according to the manufacturer’s instructions. Samples were diluted to a final concentration of 10 ng/µL. TaqMan was performed in triplicate using 20 ng of cDNA and the following TaqMan® probe (TaqMan® Gene Expression Assay): *FGFR4* (Hs01106908_m1), *FGFR1* (Hs00915142_m1), *ZEB1* (Hs00232783_m1), *SNAI1* (Hs00195591_m1), *CDH1* (Hs01023894_m1), *TWIST1* (Hs01675818_s1), *KRAS* (Hs00364284_g1), and *HNF1A* (Hs00167041_m1). *HPRT1* (Hs02800695_m1) was used as reference gene. Relative gene expression quantification was performed using the ΔΔCt method with the Sequence Detection Systems Software, Version 1.9.1 (Applied Biosystems).

**RNA sequencing**

RNA was extracted from PDAC monolayer cultures and organoids using TRIzol (Life Technologies), followed by column-based purification with the PureLink RNA Mini Kit (Ambion). The quality of purified RNA samples was determined using a Bioanalyzer 2100 (Agilent) with an RNA 6000 Nano Kit. RNAs with RNA Integrity Number (RIN) values greater than 8 were used to generate sequencing libraries using the TruSeq sample Prep Kit V2 (Illumina) according to the manufacturer’s instructions. RNA-Seq libraries prepared from organoids (n = 5) and primary cell cultures (n = 3) were multiplexed and sequenced using a NextSeq 500 platform with paired-end reads of 150 bases with a final coverage of 30 million reads per sample. RNA-Seq libraries prepared from established cell cultures (n = 20) were multiplexed and sequences using a NextSeq 500 platform with paired-end reads of 75 bases with a coverage of 3 million reads per sample. After quality control and adaptor trimming, RNA-seq reads were aligned to the GRCh38 genome build using Salmon v1.4.0[13]. Then, transcripts quantification was imported in R through tximport package v4.0[14]. Finally, count data was normalized using the R/Biocondutor package DESeq2 v1.30.0[15] in order to produce an integrated matrix of expression values. This matrix was used for all downstream analyses. Differentially expression analysis has been performed using DESeq2[15]. Gene set enrichment analysis (GSEA) was performed with *fgsea* R package v1.16.0[16] and *enrichR* package v3.0[17] using the list of differentially expressed genes sorted by log2 of fold change. Pathways are from the MSigDB database and in particular Gene Ontology, KEGG, Biocarta, Reactome and Hallmark gene sets. *fgsea* function was used with default parameters. Results were considered significant for FDR < 0.05. GSVA R package v1.38.2[18] was used to calculate the main PDAC transcriptomics subtypes (Bailey et. Al.[5], and Moffitt et al.[19]) gene set scores. *Gsva* function was used with ssgsea and gene set scores were compared among knockdown and control samples with Wilcoxon rank-sum test.

**Single-cell RNA-Sequencing**

For the integration of scRNA-seq datasets from PDAC tissues, we used the Harmony algorithm[20] via the *R* package *harmony* in order to account for the technical differences of the four datasets. The datasets (Peng et al.[9] (primary PDAC = 24, ncells = 41964), Lin et al.[21] (primary PDAC = 10, ncells = 7752), Chan-Seng-Yue et al.[4] (primary PDAC = 13, ncells = 33970) and Steele et al.[22] (primary PDAC = 16, ncells = 42844)) were first preprocessed individually using Seurat[23] for quality control and filtering (percent_mt_max = 20, nFeature_min = 500, nCount_min = 500, nCount_max = 50000) and then integrated using *harmony* function with default parameters and grouping by dataset variable. Cells were annotated with *singleR* package using the preloaded dataset *HPCA* from the *celldex* package[24] to stratify gene expression by cell population. For the normal pancreas scRNA-seq datasets, we used the integration pipeline implemented in the Seurat *R* package[25] with default parameters. The three dataset Muraro et al.[1] (ncells=2285), Segerstolpe et al.[3] (cells = 2394) and Grün et al.[2] (ncells = 1004) were downloaded from the Seurat V3 repository together with their annotation metadata. The preprocessing of the datasets counts data is described in Stuart et al.[25]; the downloaded datasets set were imported and managed through the *R* package Seurat V4.0.1[23].

**Statistical Analysis and Data mining**

For data mining and pancreatic cancer subtypes stratifications we used different gene expression datasets. The two main datasets were the PACA-AU cohort of the ICGC consortium and the TCGA-PAAD cohort of the TCGA consortium. The first was downloaded from the supplementary material of the corresponding publication[5]. This dataset contains normalized expression values (TMM normalized using edgeR Bioconductor package, converted to CPM and log2 transformed) of 96 pancreatic cancer patients. For subtypes stratification, Z-scores were calculated for each gene. Associated clinical data were downloaded from https://dcc.icgc.org/releases/current/Projects/PACA-AU. The second dataset represents the TCGA-PAAD cohort[6], downloaded from http://firebrowse.org/?cohort=PAAD, which consists of the RNA-Seq gene expression profile of 178 pancreatic cancer patients. According to other publications that disputed the histologies of some samples, we restricted the samples’ number to 148. Additional datasets included: PanCuRx[4] (EGA archive accession [EGAS00001002543](https://www.ebi.ac.uk/ega/studies/EGAS00001002543)), Moffitt et al.[19] (GEO accession: GSE71729), and Cancer Cell line Encyclopedia (CCLE)[26]. PanCuRx dataset was preprocessed with STAR v2.7.6a[27] and RSEM v1.3.1[28] and eventually a vst expression matrix was produced. For Moffitt dataset, we downloaded the matrix of log2-transformed background-corrected Cy5 microarray signal, whereas for CCLE cohort we downloaded the TPM matrix. For harmonization, Z-scores standardization was independently performed for each dataset before subsequent analysis. Expression of *FGFR1* and *FGFR4* were stratified according to tumour stage, subtype classification and survival status. The correlation of FGFR1 and FGFR4 expression with other genes was evaluated using Spearman’s correlation test (significant p-value < 0.001). To classify tumours from the PanCuRx and the ICGC datasets according to the expression of *FGFR1*, *FGFR4*, and GATA6, the distribution of their transcripts levels in the respective cohorts was assessed. We observed a bimodal and a normal distribution in the PanCuRx and in the ICGC, respectively. Samples from Bailey et al. were therefore stratified according to the median value of *FGFR1,* *FGFR4,* and *GATA6* expression. In the PanCuRx dataset, the following thresholds were used for *FGFR1* *FGFR4*, and *GATA6*, respectively: < 3.3, < 3.4, and 3.5 vst. Survival analysis has been performed through the R packages survival v3.2.10 (<https://CRAN.R-project.org/package=survival>) and the graphical representation done with survminer v0.4.9 (https://CRAN.R-project.org/package=survival). We integrated the levels of *FGFR4* and *FGFR1* expression with follow-up information for patients and drew Kaplan-Meyer curves for survival stratifying the results according to mean, median, 1st and 3rd quartiles. GraphPad Prism was used for graphical representation of data. Unless indicated, all the p-values refer to Student t test.

**ChIP-Seq and DNA Methylation datasets**

We analyzed ChIP-seq data from two different datasets: Kalisz et al.[29], which included H3K27ac*,* H3K27me3, and HNF1A ChIP-seq for mouse pancreatic cells proficient and deficient for HNF1A and all integrated with expression data; Diaferia et al.[7], which included H3K27ac*,* H3K4me1, H3K4me3, and H3K9me9 ChIP-Seq data and corresponding RNA-seq data for low grade and high grade PDAC cells. In both cases, data were preprocessed as described in the referenced papers with the only difference that Diaferia et al. dataset was re-aligned to GRCh38 human genome build. DNA methylation data were retrieved from Nones et al.[30] The methylation score for *FGFR4* was eventually compared to expression data and stratified according to the Bailey et al.[5] subtype classification

**REFERENCES TO SUPPLEMENTAL MATERIAL**

1 Muraro MJ, Dharmadhikari G, Grun D, Groen N, Dielen T, Jansen E *et al*. A Single-Cell Transcriptome Atlas of the Human Pancreas. *Cell Syst* 2016; 3: 385-394 e383.

2 Grun D, Muraro MJ, Boisset JC, Wiebrands K, Lyubimova A, Dharmadhikari G *et al*. De Novo Prediction of Stem Cell Identity using Single-Cell Transcriptome Data. *Cell Stem Cell* 2016; 19: 266-277.

3 Segerstolpe A, Palasantza A, Eliasson P, Andersson EM, Andreasson AC, Sun X *et al*. Single-Cell Transcriptome Profiling of Human Pancreatic Islets in Health and Type 2 Diabetes. *Cell Metab* 2016; 24: 593-607.

4 Chan-Seng-Yue M, Kim JC, Wilson GW, Ng K, Figueroa EF, O'Kane GM *et al*. Transcription phenotypes of pancreatic cancer are driven by genomic events during tumor evolution. *Nat Genet* 2020; 52: 231-240.

5 Bailey P, Chang DK, Nones K, Johns AL, Patch AM, Gingras MC *et al*. Genomic analyses identify molecular subtypes of pancreatic cancer. *Nature* 2016; 531: 47-52.

6 Cancer Genome Atlas Research Network. Electronic address aadhe, Cancer Genome Atlas Research N. Integrated Genomic Characterization of Pancreatic Ductal Adenocarcinoma. *Cancer Cell* 2017; 32: 185-203 e113.

7 Diaferia GR, Balestrieri C, Prosperini E, Nicoli P, Spaggiari P, Zerbi A *et al*. Dissection of transcriptional and cis-regulatory control of differentiation in human pancreatic cancer. *EMBO J* 2016; 35: 595-617.

8 Somerville TDD, Xu Y, Miyabayashi K, Tiriac H, Cleary CR, Maia-Silva D *et al*. TP63-Mediated Enhancer Reprogramming Drives the Squamous Subtype of Pancreatic Ductal Adenocarcinoma. *Cell Rep* 2018; 25: 1741-1755 e1747.

9 Peng J, Sun BF, Chen CY, Zhou JY, Chen YS, Chen H *et al*. Single-cell RNA-seq highlights intra-tumoral heterogeneity and malignant progression in pancreatic ductal adenocarcinoma. *Cell Res* 2019; 29: 725-738.

10 Boj SF, Hwang CI, Baker LA, Chio, II, Engle DD, Corbo V *et al*. Organoid models of human and mouse ductal pancreatic cancer. *Cell* 2015; 160: 324-338.

11 Huch M, Bonfanti P, Boj SF, Sato T, Loomans CJ, van de Wetering M *et al*. Unlimited in vitro expansion of adult bi-potent pancreas progenitors through the Lgr5/R-spondin axis. *EMBO J* 2013; 32: 2708-2721.

12 Olive KP, Jacobetz MA, Davidson CJ, Gopinathan A, McIntyre D, Honess D *et al*. Inhibition of Hedgehog signaling enhances delivery of chemotherapy in a mouse model of pancreatic cancer. *Science* 2009; 324: 1457-1461.

13 Patro R, Duggal G, Love MI, Irizarry RA, Kingsford C. Salmon provides fast and bias-aware quantification of transcript expression. *Nat Methods* 2017; 14: 417-419.

14 Soneson C, Love MI, Robinson MD. Differential analyses for RNA-seq: transcript-level estimates improve gene-level inferences. *F1000Res* 2015; 4: 1521.

15 Love MI, Huber W, Anders S. Moderated estimation of fold change and dispersion for RNA-seq data with DESeq2. *Genome Biol* 2014; 15: 550.

16 Subramanian A, Tamayo P, Mootha VK, Mukherjee S, Ebert BL, Gillette MA *et al*. Gene set enrichment analysis: a knowledge-based approach for interpreting genome-wide expression profiles. *Proc Natl Acad Sci U S A* 2005; 102: 15545-15550.

17 Kuleshov MV, Jones MR, Rouillard AD, Fernandez NF, Duan Q, Wang Z *et al*. Enrichr: a comprehensive gene set enrichment analysis web server 2016 update. *Nucleic Acids Res* 2016; 44: W90-97.

18 Hanzelmann S, Castelo R, Guinney J. GSVA: gene set variation analysis for microarray and RNA-seq data. *BMC Bioinformatics* 2013; 14: 7.

19 Moffitt RA, Marayati R, Flate EL, Volmar KE, Loeza SG, Hoadley KA *et al*. Virtual microdissection identifies distinct tumor- and stroma-specific subtypes of pancreatic ductal adenocarcinoma. *Nat Genet* 2015; 47: 1168-1178.

20 Korsunsky I, Millard N, Fan J, Slowikowski K, Zhang F, Wei K *et al*. Fast, sensitive and accurate integration of single-cell data with Harmony. *Nat Methods* 2019; 16: 1289-1296.

21 Lin W, Noel P, Borazanci EH, Lee J, Amini A, Han IW *et al*. Single-cell transcriptome analysis of tumor and stromal compartments of pancreatic ductal adenocarcinoma primary tumors and metastatic lesions. *Genome Med* 2020; 12: 80.

22 Steele NG, Carpenter ES, Kemp SB, Sirihorachai V, The S, Delrosario L *et al*. Multimodal Mapping of the Tumor and Peripheral Blood Immune Landscape in Human Pancreatic Cancer. *Nat Cancer* 2020; 1: 1097-1112.

23 Hao Y, Hao S, Andersen-Nissen E, Mauck WM, 3rd, Zheng S, Butler A *et al*. Integrated analysis of multimodal single-cell data. *Cell* 2021; 184: 3573-3587 e3529.

24 Aran D, Looney AP, Liu L, Wu E, Fong V, Hsu A *et al*. Reference-based analysis of lung single-cell sequencing reveals a transitional profibrotic macrophage. *Nat Immunol* 2019; 20: 163-172.

25 Stuart T, Butler A, Hoffman P, Hafemeister C, Papalexi E, Mauck WM, 3rd *et al*. Comprehensive Integration of Single-Cell Data. *Cell* 2019; 177: 1888-1902 e1821.

26 Barretina J, Caponigro G, Stransky N, Venkatesan K, Margolin AA, Kim S *et al*. The Cancer Cell Line Encyclopedia enables predictive modelling of anticancer drug sensitivity. *Nature* 2012; 483: 603-607.

27 Dobin A, Davis CA, Schlesinger F, Drenkow J, Zaleski C, Jha S *et al*. STAR: ultrafast universal RNA-seq aligner. *Bioinformatics* 2013; 29: 15-21.

28 Li B, Dewey CN. RSEM: accurate transcript quantification from RNA-Seq data with or without a reference genome. *BMC Bioinformatics* 2011; 12: 323.

29 Kalisz M, Bernardo E, Beucher A, Maestro MA, Del Pozo N, Millan I *et al*. HNF1A recruits KDM6A to activate differentiated acinar cell programs that suppress pancreatic cancer. *EMBO J* 2020; 39: e102808.

30 Nones K, Waddell N, Song S, Patch AM, Miller D, Johns A *et al*. Genome-wide DNA methylation patterns in pancreatic ductal adenocarcinoma reveal epigenetic deregulation of SLIT-ROBO, ITGA2 and MET signaling. *Int J Cancer* 2014; 135: 1110-1118.
