# Supplementary figures and images for: Loss of FGFR4 promotes the malignant phenotype of PDAC
Source: Oncogene. 2022 Aug 13;41(38):4371–84. doi: 10.1038/s41388-022-02432-5 (PMC9481460; doi:10.1038/s41388-022-02432-5)

**A**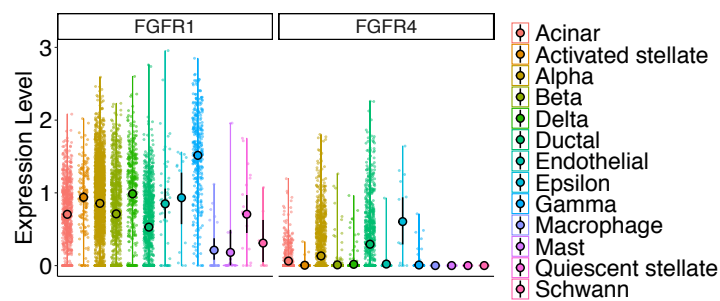**B**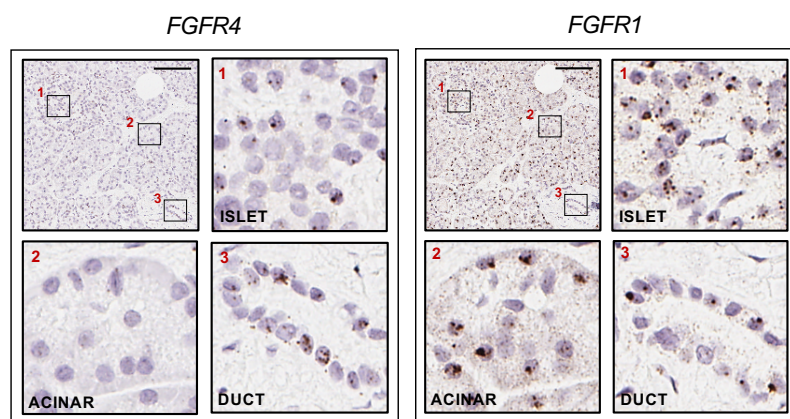**C**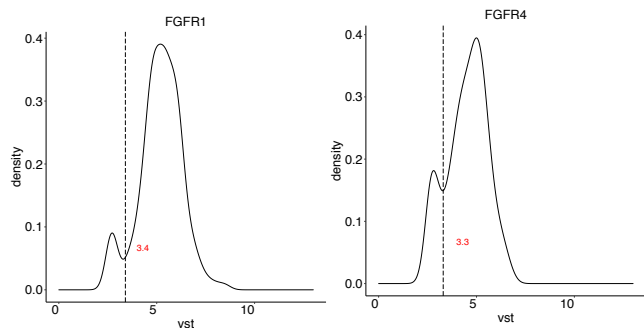**D**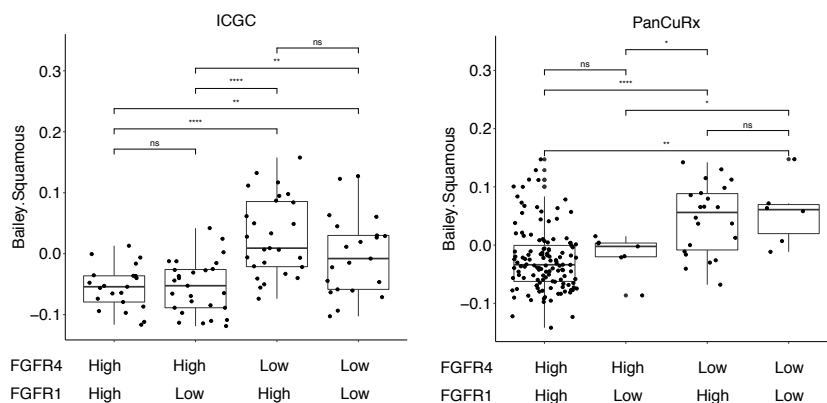**E**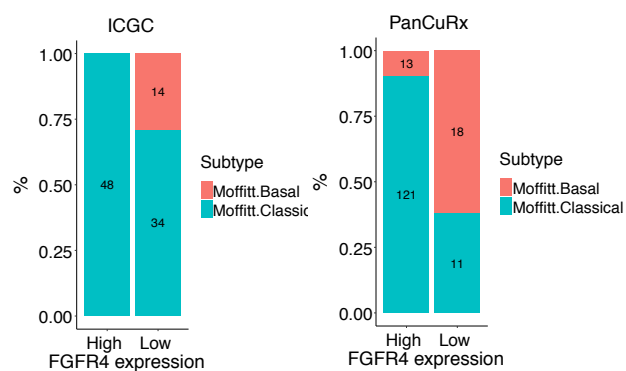**F**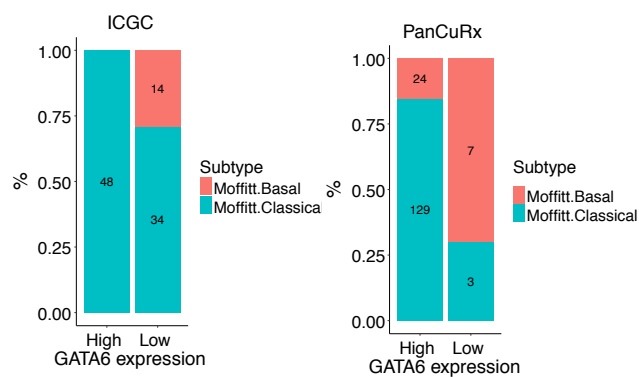**G**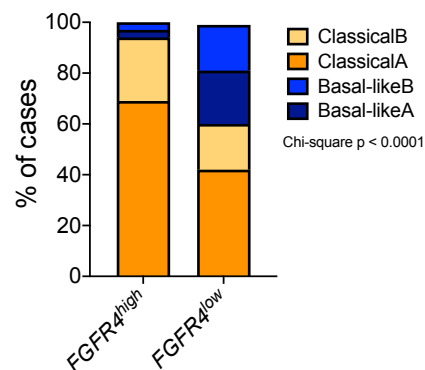

Supplement: Supplementary file 2 — Supplemental Figure 1 [file 41388_2022_2432_MOESM2_ESM.pdf]

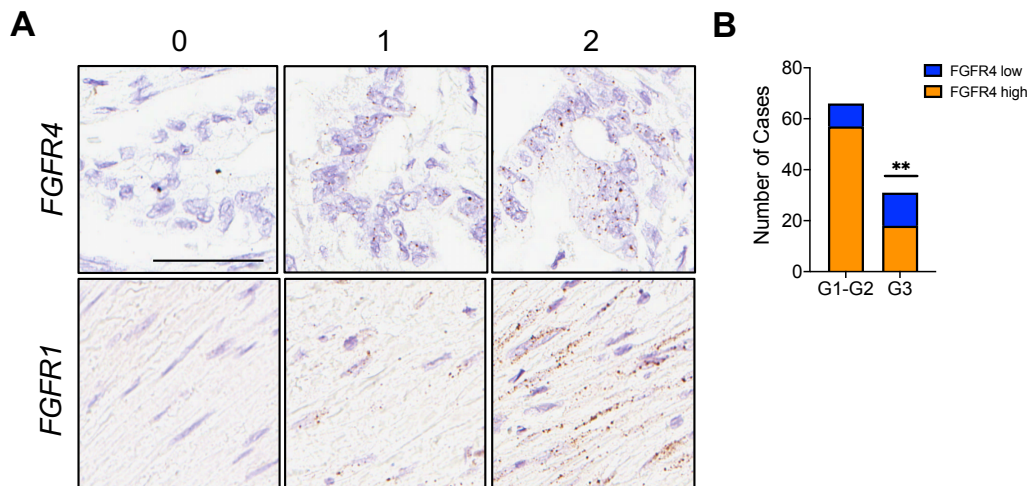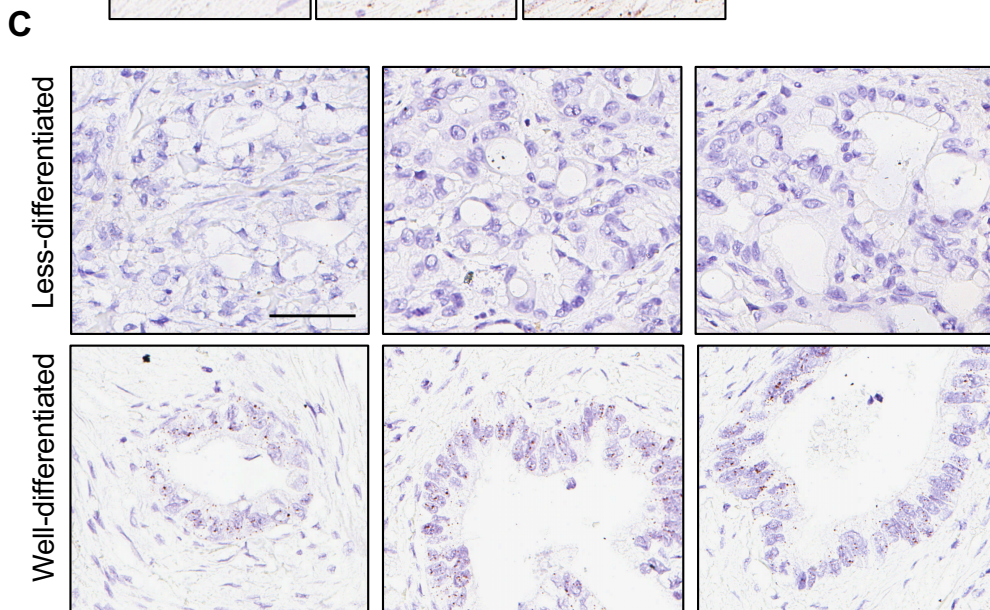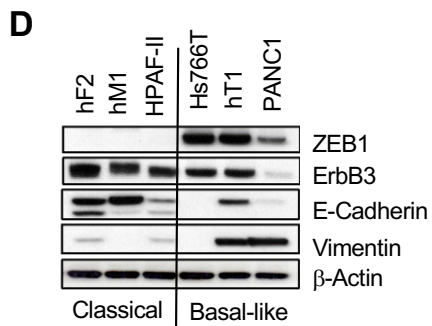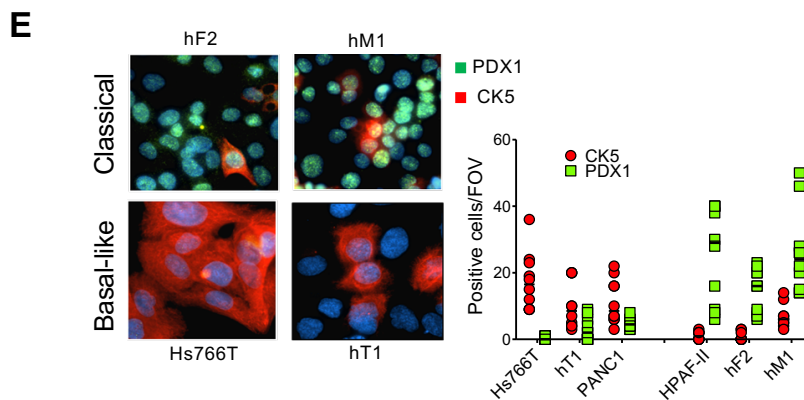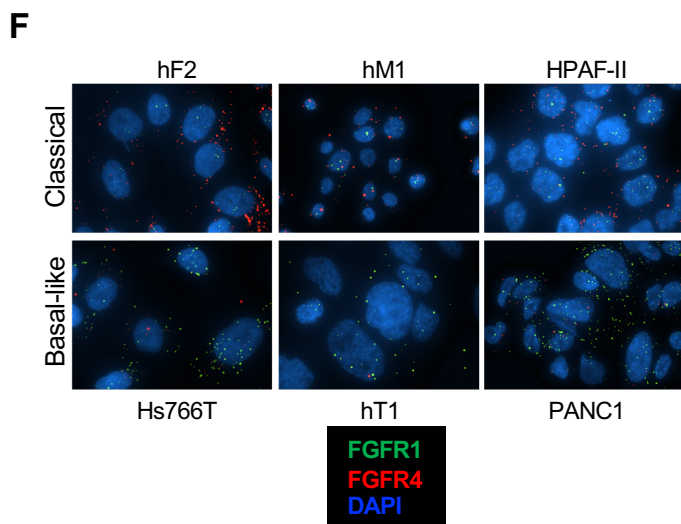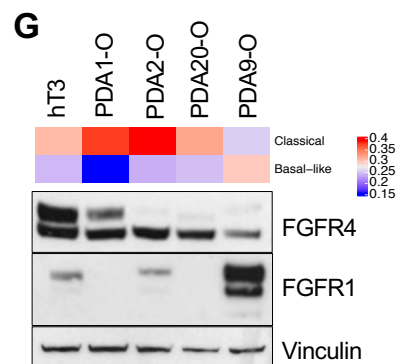

Supplement: Supplementary file 3 — Supplemental Figure 2 [file 41388_2022_2432_MOESM3_ESM.pdf]

**A**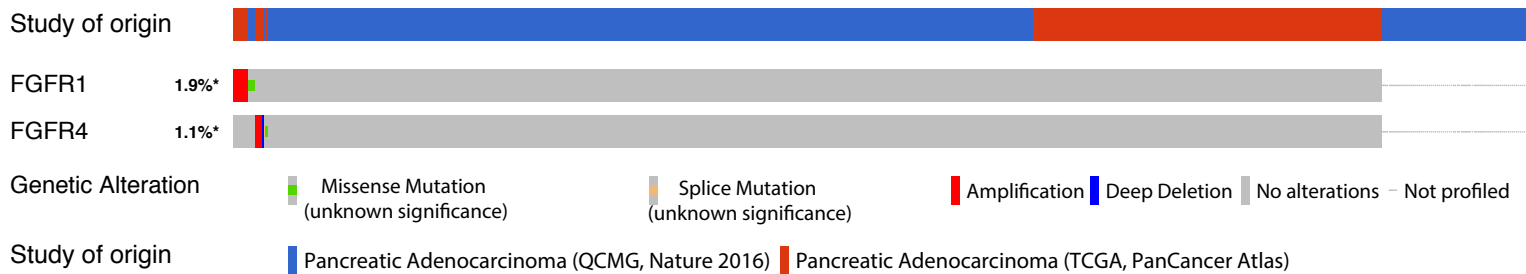**B**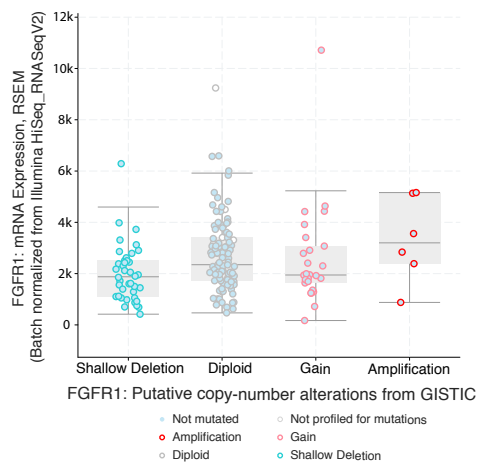**C**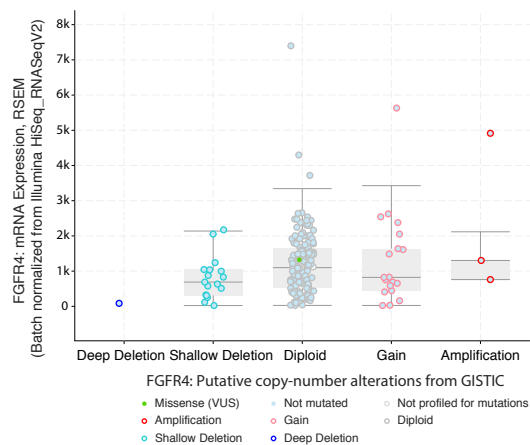**D**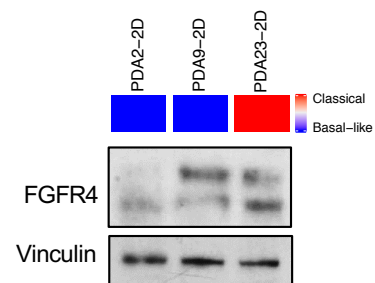**E**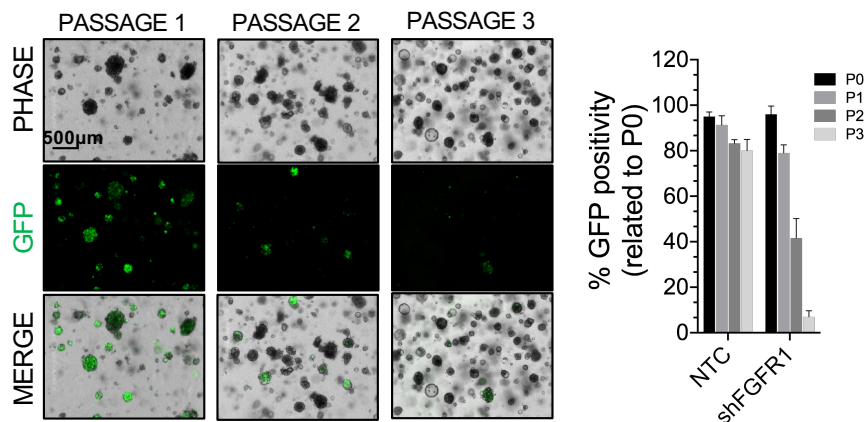**F**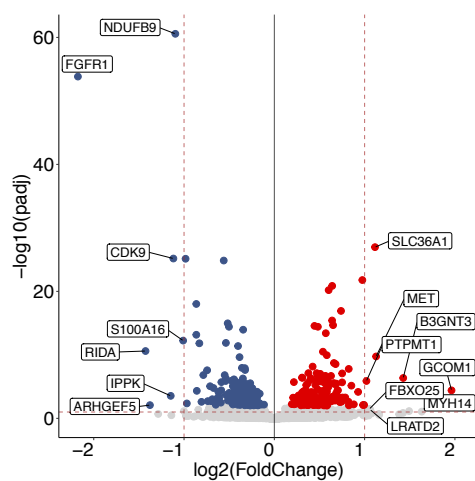**G**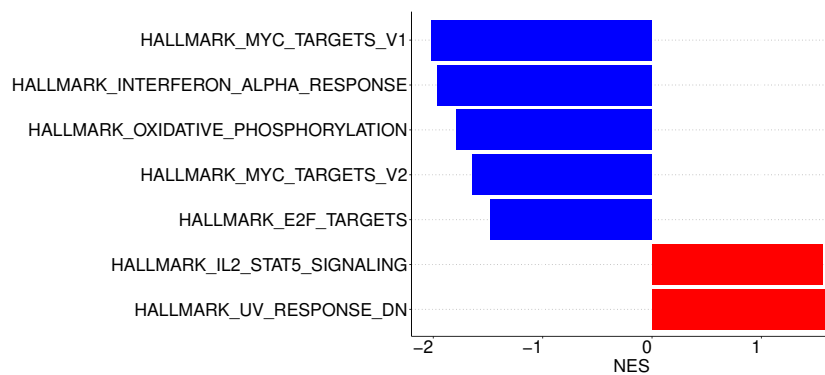

Supplement: Supplementary file 4 — Supplemental Figure 3 [file 41388_2022_2432_MOESM4_ESM.pdf]

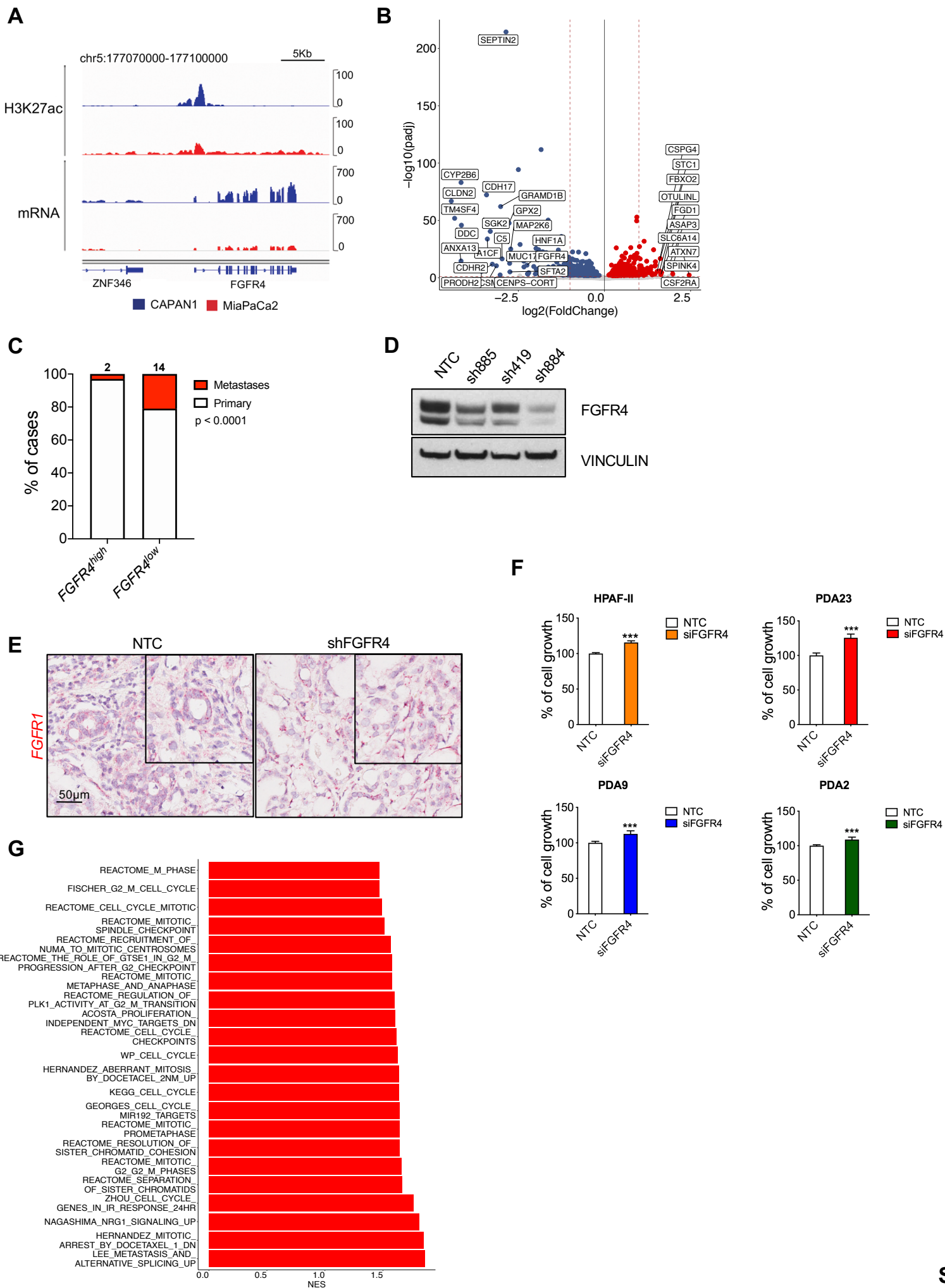

Supplement: Supplementary file 5 — Supplemental Figure 4 [file 41388_2022_2432_MOESM5_ESM.pdf]

**A**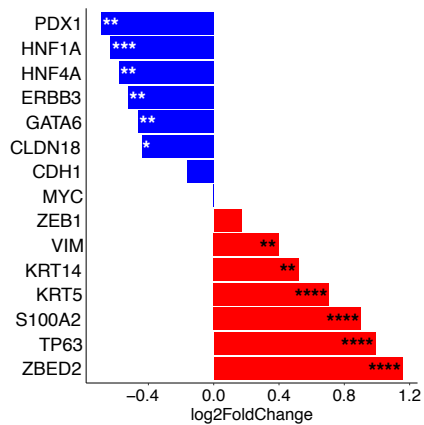**B**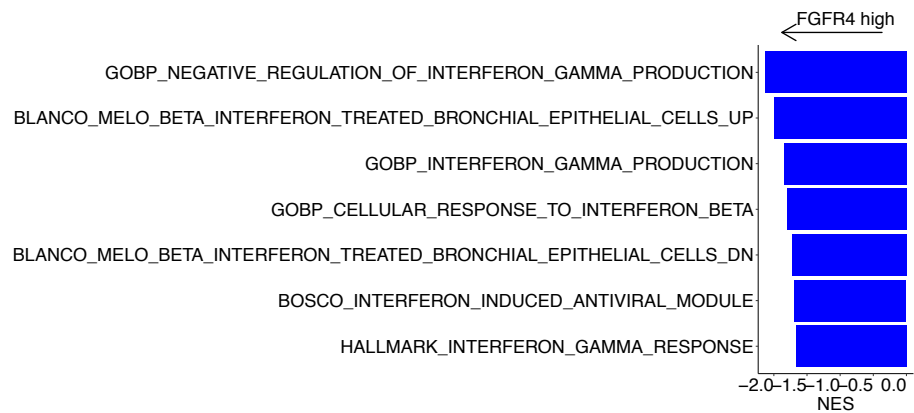**C**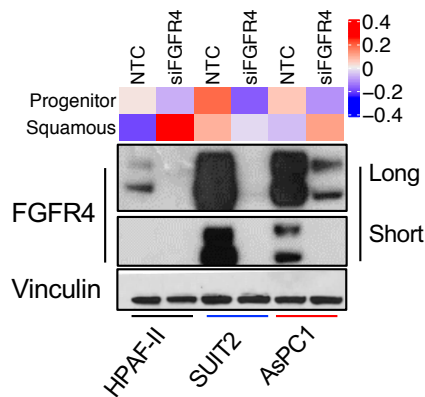**D**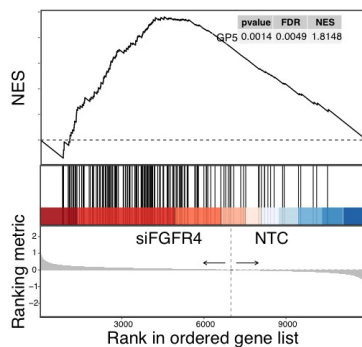**E**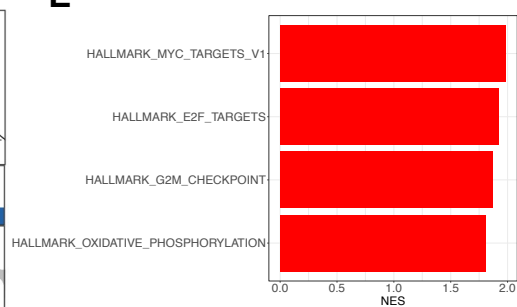**F**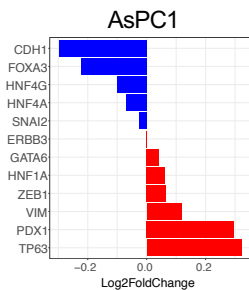**G**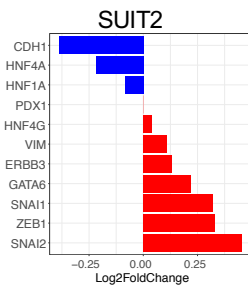**H**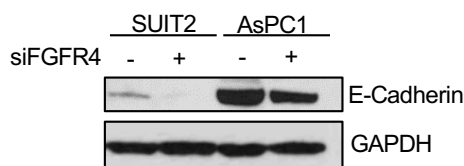**I**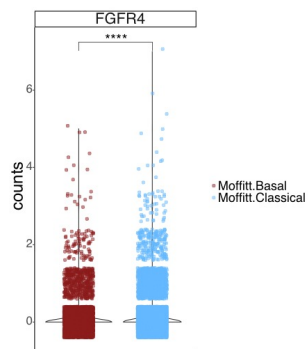**J**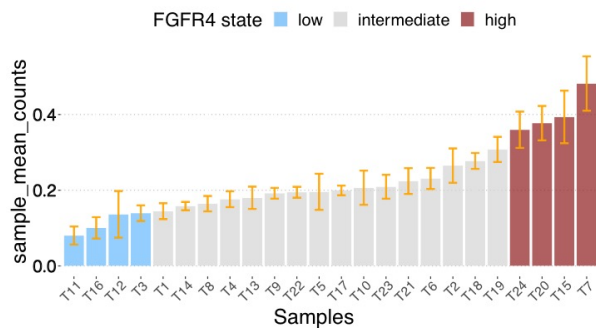**K**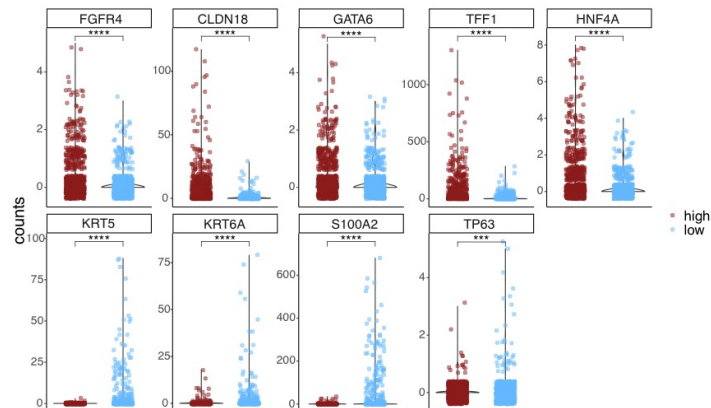

Supplement: Supplementary file 6 — Supplemental Figure 5 [file 41388_2022_2432_MOESM6_ESM.pdf]

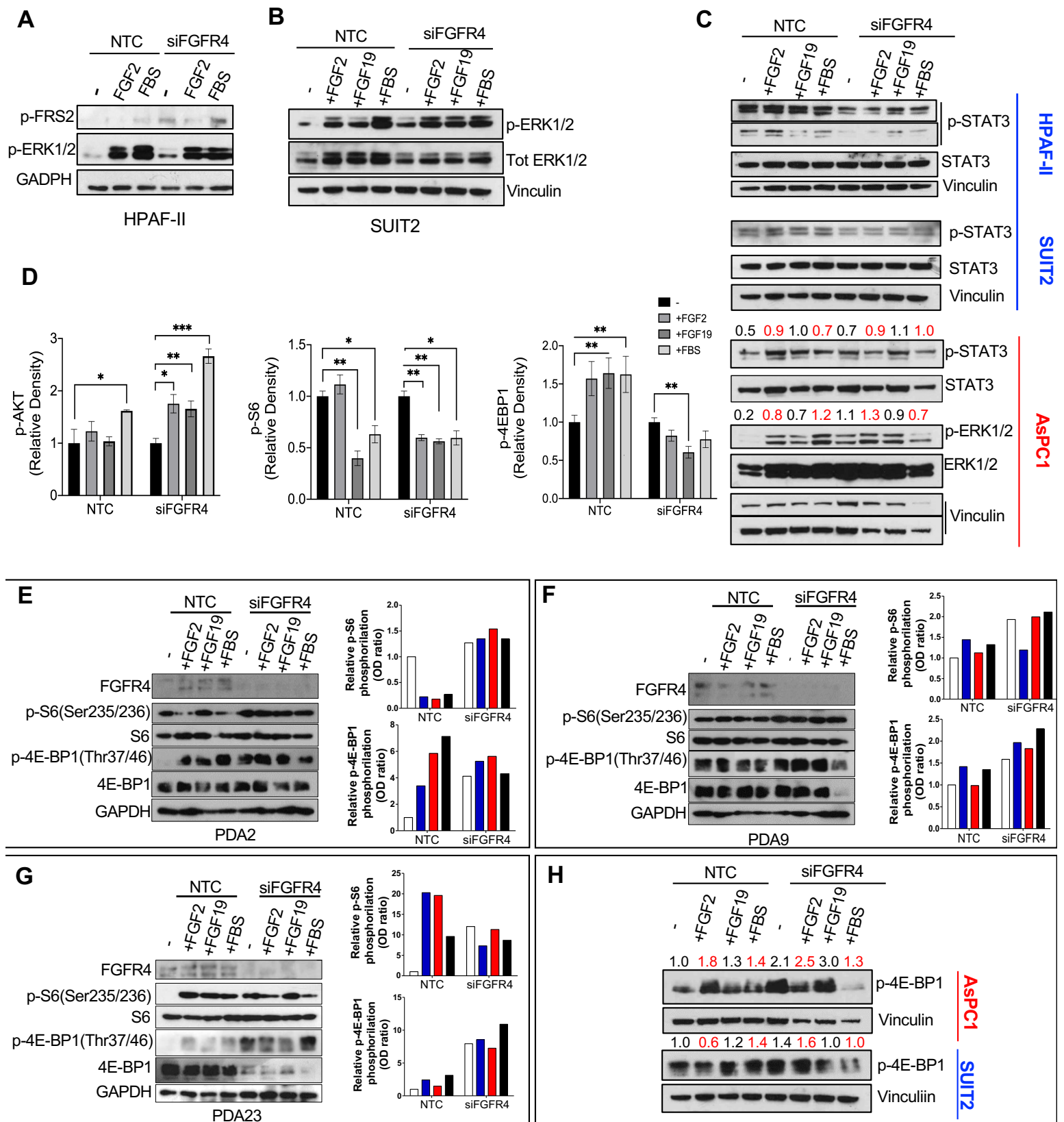

Supplement: Supplementary file 7 — Supplemental Figure 6 [file 41388_2022_2432_MOESM7_ESM.pdf]
